# Magneto‐Orientation of Magnetic Double Stacks for Patterned Anisotropic Hydrogels with Multiple Responses and Modulable Motions

**DOI:** 10.1002/anie.202207272

**Published:** 2022-07-14

**Authors:** Chen Fei Dai, Olena Khoruzhenko, Chengqian Zhang, Qing Li Zhu, Dejin Jiao, Miao Du, Josef Breu, Peng Zhao, Qiang Zheng, Zi Liang Wu

**Affiliations:** ^1^ Ministry of Education Key Laboratory of Macromolecular Synthesis and Functionalization Department of Polymer Science and Engineering Zhejiang University Hangzhou 310027 China; ^2^ Bavarian Polymer Institute and Department of Chemistry University of Bayreuth Universitätsstrasse 30 95440 Bayreuth Germany; ^3^ The State Key Laboratory of Fluid Power Transmission and Control Systems Key Laboratory of 3D Printing Process and Equipment of Zhejiang Province School of Mechanical Engineering Zhejiang University Hangzhou 310028 China

**Keywords:** Anisotropic Hydrogels, Ferronematic Liquid Crystals, Magnetic Orientation, Nanosheets, Soft Robots

## Abstract

Reported here is a multi‐response anisotropic poly(*N*‐isopropylacrylamide) hydrogel developed by using a rotating magnetic field to align magnetic double stacks (MDSs) that are fixed by polymerization. The magneto‐orientation of MDSs originates from the unique structure with γ‐Fe_2_O_3_ nanoparticles sandwiched by two silicate nanosheets. The resultant gels not only exhibit anisotropic optical and mechanical properties but also show anisotropic responses to temperature and light. Gels with complex ordered structures of MDSs are further devised by multi‐step magnetic orientation and photolithographic polymerization. These gels show varied birefringence patterns with potentials as information materials, and can deform into specific configurations upon stimulations. Multi‐gait motions are further realized in the patterned gel through dynamic deformation under spatiotemporal light and friction regulation by imposed magnetic force. The magneto‐orientation assisted fabrication of hydrogels with anisotropic structures and additional functions should bring opportunities for gel materials in biomedical devices, soft actuators/robots, etc.

## Graphical abstract

Multi‐response anisotropic hydrogels are developed by using a rotating magnetic field to orient the magnetic double stacks (MDSs) with γ‐Fe_2_O_3_ nanoparticles sandwiched by two nanosheets. Patterned gels with sophisticated orientations of MDSs are fabricated by multi‐step magnetic orientation and photolithographic polymerization, which exhibit programmed deformations and motions under synergetic actuations.

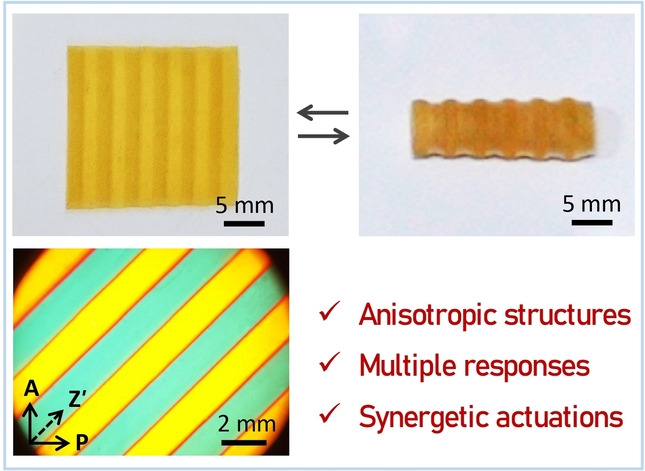

## Introduction

Concepts that draw inspirations from biological tissues have enabled substantial progress in creating artificial intelligent materials.^[1]^ Hydrogels, a class of soft materials with water as the major component, have close similarity to living tissues and therefore receive particular research interest.^[2]^ Although great efforts have been made to improve the mechanical properties to match specific soft tissues,^[3]^ synthetic hydrogels usually have an isotropic network, different from the anisotropic structures of biological tissues such as muscles and ligaments.[^4^, ^5^] Creating ordered structures in hydrogel materials should afford them with anisotropic optical, mechanical, and response properties, thereby promoting their applications in biomedical and engineering fields.

In recent years, various anisotropic materials have been developed by forming ordered structures of polymer chains or inorganic fillers in the soft matrices.[^5^, ^6^] One straightforward strategy is to orient polymer chains by stretching the materials and then fixing this anisotropic structure;^[7]^ but this method requires the material capable of withstanding considerable deformation and force, and can only produce materials with monodomain anisotropic structures. Another strategy is to align rigid molecules or anisotropic fillers under external fields and then freeze the ordered structures by curing of the precursors.[^8^, ^9^, ^10^, ^11^, ^12^, ^13^, ^14^] For example, Tovar and co‐workers used shear forces to align π‐conjugated peptide nanostructures in hydrogels that showed directionally dependent optical signal and electrical response.^[8a]^ Anisotropic hydrogels can also be obtained by electric field‐induced orientation of anisotropic particles with the permanent or induced dipole moment.^[9]^ However, the direct contact with precursor solutions may result in unexpected electrochemical reactions and/or electrophoresis during gel synthesis. Different from the aforementioned strategies, magnetic fields can be applied in non‐contact mode to orient various mesogens and inorganic fillers in order to fabricate anisotropic materials.[^10^, ^11^, ^12^, ^13^, ^14^] For instance, Stupp et al. developed anisotropic hydrogels with the assistance of a static magnetic field to orient nickel nanowires that were embedded in a photoresponsive polymer matrix; the resultant hydrogels were used as continuum soft robots and showed multi‐gait motions steered by light and magnetic field.^[11]^ However, it is still a grand challenge to develop patterned hydrogels with sophisticated anisotropic structures.

Besides nanoparticles and nanowires, two‐dimensional (2D) nanosheets can also be oriented by magnetic fields to form lamella‐like ordered structures that will further enhance the anisotropic properties and responses of the resultant hydrogels.[^12^, ^13^, ^14^] For example, Studart et al. decorated Al_2_O_3_ platelets with Fe_3_O_4_ nanoparticles and then aligned them by a rotating magnetic field to develop anisotropic poly(*N*‐isopropylacrylamide) (PNIPAm) hydrogels. Bilayer hydrogel was further prepared by multi‐step magnetic orientation and polymerization to form sophisticated anisotropic structures of the platelets, which showed programmed deformations under external stimuli.^[13a]^ Another seminal work by Aida et al. reported on the development of muscle‐like anisotropic hydrogels applied an ultrastrong (≈10 T) superconducting magnetic field to perfectly align titanate nanosheets.^[14]^ The obtained nanocomposite PNIPAm hydrogel exhibited fast and isochoric deformations upon heating due to the reversibly changes of electrostatic permittivity and the resulting anisotropic electrostatic repulsion. They further used this hydrogel to realize earthworm‐like peristaltic crawling in a confined glass tube upon periodic light irradiation after incorporating gold nanoparticles.^[14b]^ However, it is still challenging to develop complex ordered structures in the hydrogel by using such a superconducting magnetic field. In addition, the nanosheets and photothermal nanoparticles are separately introduced into the hydrogel to afford photo‐induced anisotropic response. The possible diffusion of the nanoparticles out of the matrix may lead to reduced performances of the gel. On the other hand, the locomotion of anisotropic gels usually relies on specific strategies to convert cyclic bending/unbending or stretching/contraction deformations into directional motions by using a rachet plate or creating asymmetric shape of the material.[^14d^, ^15^] Programmable locomotion with switchable direction and gait mode has been rarely realized in anisotropic gels, which require sophisticated controls of the distributed structures and cooperative actuations for dynamic morphing.^[1c]^ Clearly, it is highly desirable to develop hydrogels with sophisticated anisotropic structures and multiple responses by applying a weak magnetic field to align the functional nanosheets, which can be coupled with other advanced fabrication technologies.

Here we report a series of anisotropic PNIPAm hydrogels fabricated by applying a weak rotating magnetic field (≈260 mT) to orient 2D magnetic double stacks (MDSs) followed by polymerization to fix the ordered structures. The MDSs have a unique structure with γ‐Fe_2_O_3_ nanoparticles being sandwiched by two silicate layers, which can be dispersed in water with high stability.^[15a]^ The aqueous suspensions show ferronematic phases at a very low content, which are responsive to magnetic field and light. The obtained monodomain PNIPAm hydrogels have anisotropic optical and mechanical properties due to the highly‐ordered arrangement of the MDSs. The hydrogels also exhibit anisotropic response upon heating or light irradiation. Hydrogels with sophisticated ordered structures of MDSs are further developed by multi‐step magnetic orientation and photolithographic polymerization, which show complex birefringent patterns and deform into three‐dimensional (3D) configurations. Versatile locomotion is realized in the patterned hydrogel by dynamic actuations under spatiotemporal light and magnetic field. The strategy of preparing anisotropic hydrogels and the light‐ and magnet‐steered locomotion should be informative for the design of functional materials and soft actuators with versatile applications.

## Results and Discussion

**Figure 1:**
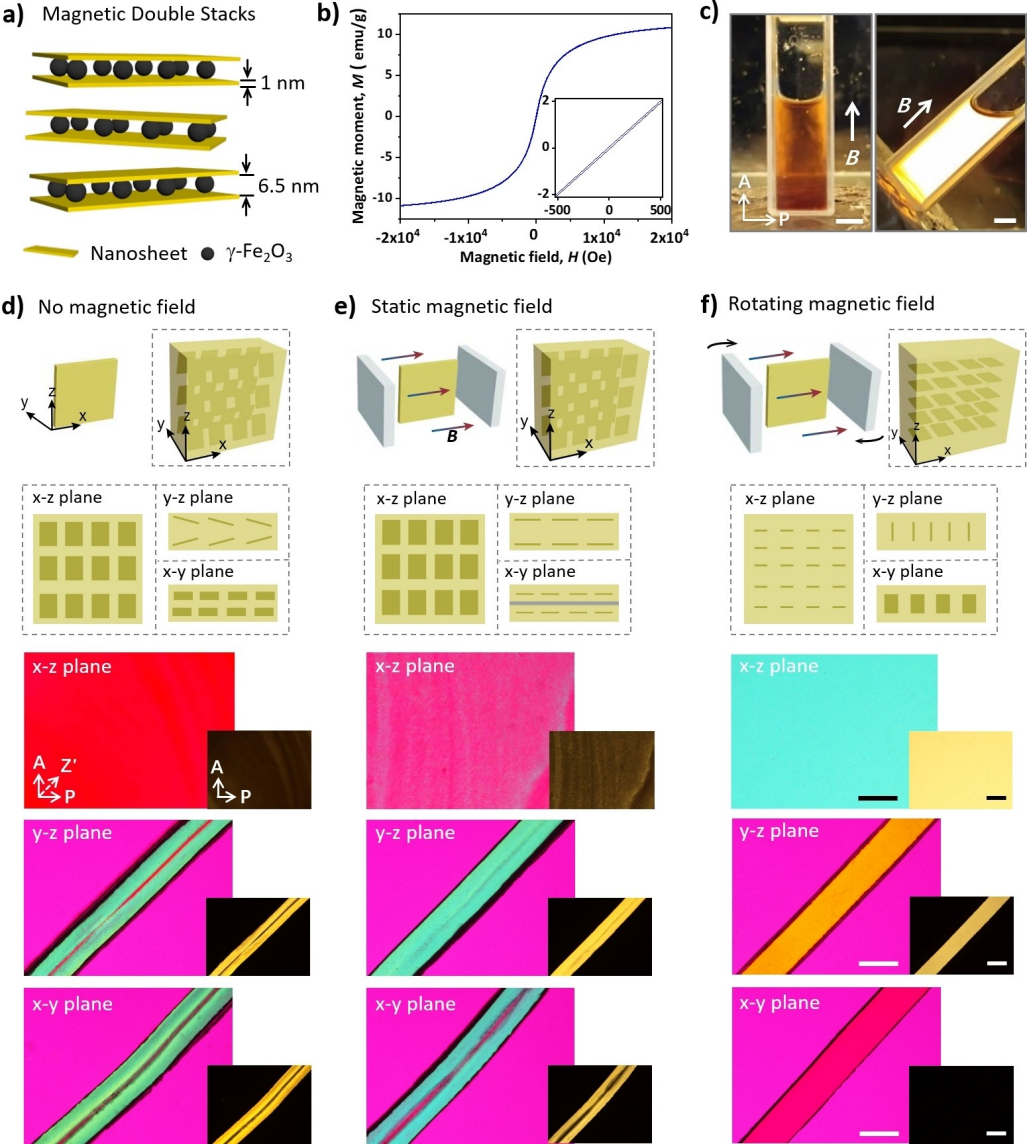
a) Schematic of the sandwich‐like structure of MDSs. b) Magnetic hysteresis loop of MDS powder. c) Photos of a ferronematic suspension of MDS (1 wt%) between a pair of crossed polarized films. A magnet is placed under the cuvette filled with the MDS suspension. Scale bar: 5 mm. d–f) Schematic for the gel synthesis and the alignments of MDSs (top) and POM images of the hydrogel sheets observed from different directions (bottom). The hydrogels were prepared without magnetic field (d), with static magnetic field (e), or with rotating magnetic field (f). A: analyzer, P: polarizer, Z′: slow axis of 530 nm tint plate. Scale bar: 1 mm.

The magnetic double stacks (MDSs) were synthesized according to a previously published procedure.^[16]^ The generic structure of MDSs is shown in Figure 1a. MDSs have a sandwich‐like structure with two nanosheets of [Li_0.25_][Mg_2.5_Li_0.5_][Si_4_]O_10_F_2_ encompassing γ‐Fe_2_O_3_ nanoparticles confined in the resulting interlayer space. The singular silicate nanosheet (thickness 1 nm) has a high aspect ratio (≈20 000) and high density of negative charges (1.1 nm^−2^), while the surface of magnetic nanoparticles bears positive charges.^[17]^ As shown in Figure S1 by atomic force microscopy (AFM), the typical diameter of MDS is ≈15 μm. As γ‐Fe_2_O_3_ nanoparticles are 5.5±1.1 nm with a low polydispersity and the two individual hectorite sheets are 2×1 nm,^[16]^ the height observed by AFM of ≈8.5 nm is in good agreement with expectations, in particular as in addition the countercations attached to the upper surface need to be taken into accounts. These structural attributes afford MDSs with structural stability and good dispersibility in water. The aqueous suspension of MDSs forms a ferronematic phase even at a very low content (≈0.3 wt%) as rotation is hampered even at separations >50 nm of individual MDSs.^[16a]^ The suspension with 1 wt% of MDS exhibits strong birefringence when placed between a pair of crossed polarizing films (Figure S2). This ferronematic phase can be further oriented by mechanical shearing (Figure S3). The relationship between the orientation direction and the birefringence color provides a straightforward way to characterize the alignment of MDSs.^[18]^ The multi‐domain nematic suspension can also be transformed into a monodomain under a magnetic field, due to the presence of magnetic γ‐Fe_2_O_3_ nanoparticles (Figure S4). The sandwiching of nanoparticles affords collective magnetic response and provides MDSs with obvious superparamagnetism at ambient temperature and thus a suspension with ultrafast response to magnetic field (Figure 1b, c and Figure S5).

Anisotropic hydrogels are prepared by using a weak magnetic field (260 mT) to orient MDSs and then fixing the ordered structure by photopolymerization to form a gel matrix. The introduction of neutral monomer, chemical crosslinker, and photo‐initiator does not influence the ferronematic phase of the suspension and the magnetic orientation of MDSs. Static or rotating magnetic fields are then applied to prepare anisotropic hydrogels, which are compared with that synthesized in the absence of magnetic field (Figure 1d–f). When prepared without magnetic field, the nanocomposite hydrogel sheet (corresponding to *x*–*z* plane) shows no birefringence when viewed from the top (*y* direction, as noted in the schematic figure) under a polarizing optical microscope (POM). However, the cross‐sections of the hydrogel show strong birefringence except the middle region, indicating the formation of anisotropic structure during the gel synthesis (Figure 1d). This unexpected structure arises from the shear‐induced orientation of MDSs when the precursor solution is injected into the reaction cell; the gradual rising of the fluid results in shear‐orientation of MDSs that are fixed by subsequent polymerization (Figure S6). Due to this reason, the cross‐sections (*x*–*y* plane and *y*–*z* plane) of the hydrogel exhibit similar birefringent pattern. A careful analysis indicates that the MDSs in the cross‐section (*y*–*z* plane) are aligned at the regions close to the glass substrate, as expected for shear orientation with an oblique angle of ±15° to the surface.

When a static magnetic field is applied, the anisotropic structure of the hydrogel is only slightly improved, as revealed by POM observations. Under static magnetic field, the MDSs previously tilted to the glass substrate are oriented along the magnetic field. No birefringence is found in the *x*–*z* plane of the hydrogel film, yet uniform birefringence is observed in the *y*–*z* and *x*–*y* planes (Figure 1e and Figure S7). This is because a static magnetic field only results in uniaxial orientation of MDSs along the magnetic field and lacks control over other directions. When a rotating magnetic field is applied, the hydrogel has, however, a much better anisotropic structure of MDSs (Figure 1f and Figure S8). The MDSs tend to follow the direction of magnetic field to minimize their magnetic energy and rotate synchronously with the rotating magnetic field. Therefore, the nanosheets align in the rotating plane of the magnetic field, i.e. parallel to the *x*–*y* plane of the sample.[^13b^, ^19^] In other words, biaxial orientation of MDSs in the suspension is achieved under the rotating magnetic field, which can be immobilized further by photopolymerization. This strategy results in a monodomain hydrogel with MDSs perfectly aligned along the planar rotating magnetic field. The anisotropic structure of hydrogel is confirmed by POM observations.

We further examine the influences of rotating frequency, action time, and strength of the rotating magnetic field on the degree of anisotropy of resultant hydrogels. As shown in Figure S9a, the hydrogels show almost identical birefringence, indicating that the rotating frequency ranging from 10 to 60 Hz has little influence on the alignment of MDSs.[^13b^, ^19^] The action time of the rotating magnetic field also shows little influence on the alignment of MDSs (Figure S9b). When the action time increases from 20 s to 10 min, the hydrogels show the same birefringence, indicating a high efficiency of rotating magnetic field to align the MDSs. As the magnetic field strength determines the magnetic torque, it is expected to be important for the magnetic orientation. As the magnetic field strength increases from 40 to 260 mT, the intensity of birefringence indeed increases, indicating an improved orientation degree of MDSs (Figure S9c). Therefore, the rotating frequency, action time, and strength of the rotating magnetic field are set as 30 Hz, 20 s and 260 mT respectively, for the synthesis of anisotropic gels.

**Figure 2:**
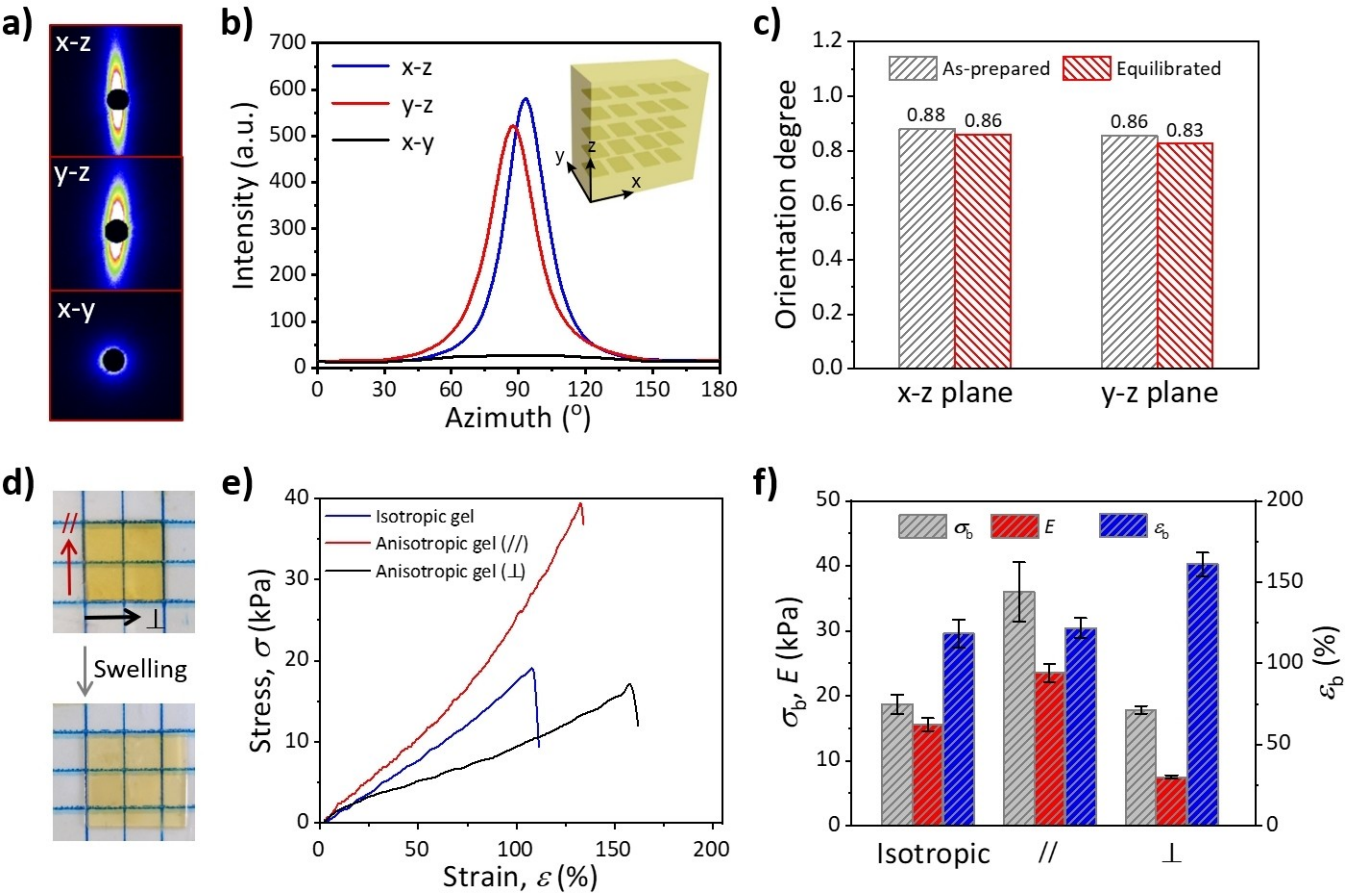
a–c) 2D SAXS patterns (a), scattering intensity–azimuth plots (b), and orientation degree of MDS (c) of the as‐prepared hydrogel. Orientation degree of MDS in the equilibrated gel is also presented in (c). d) Photos of the anisotropic hydrogel before and after the swelling process. e) Tensile stress–strain curves of the isotropic gel and anisotropic gel stretched from // and ⊥ directions. f) Corresponding mechanical parameters of the gels, including Young's modulus *E*, breaking stress *σ*_b_, and breaking strain ϵ_b_. Error bars represent the standard deviation of the mean (*n*=3).

The alignment of MDSs endows the hydrogel with anisotropic optical and mechanical properties.[^12a^, ^19^] Strong anisotropic SAXS patterns are observed in the *x*–*z* and *y*–*z* planes of the as‐prepared gel, whereas an isotropic pattern is found in the *x*–*y* plane (Figure 2a–c), which further confirms the alignments of MDSs in the gel. The calculated orientation degree of MDSs in the as‐prepared gel is 0.88, which slightly decreases to 0.86 after being equilibrated in water due to the weak volume expansion of the gel (Figure S10). The swelling ratio in length of the hydrogel is also anisotropic, i.e. 1.2 and 1.3 in the direction parallel (//) and perpendicular (⊥ ) to the alignment of MDSs (Figure 2d and Figure S11). This hydrogel also shows anisotropic mechanical properties. As shown in Figure 2e and 2f, the Young's modulus of the gel stretched along the // direction is ≈3 times that along the ⊥ direction, while the breaking strain along the // direction is ≈40 % lower than that along the ⊥ direction. This anisotropic mechanical property arises from the highly‐ordered reinforcing MDSs. Rigid MDSs as the stiff crosslinking junctions restrict the in‐plane deformation of the gel matrix, thereby resulting in larger modulus and strength of the hydrogel along the // direction.[^9b^, ^20^] An isotropic hydrogel containing randomly dispersed MDSs is prepared (Figure S12, see experiment details in Supporting Information) for comparison of the mechanical properties with those of the anisotropic gel. It is rational that the modulus of the MDS‐containing isotropic gel is between the moduli of the anisotropic gel in the // direction and ⊥ direction (Figure 2e).^[14a]^

**Figure 3:**
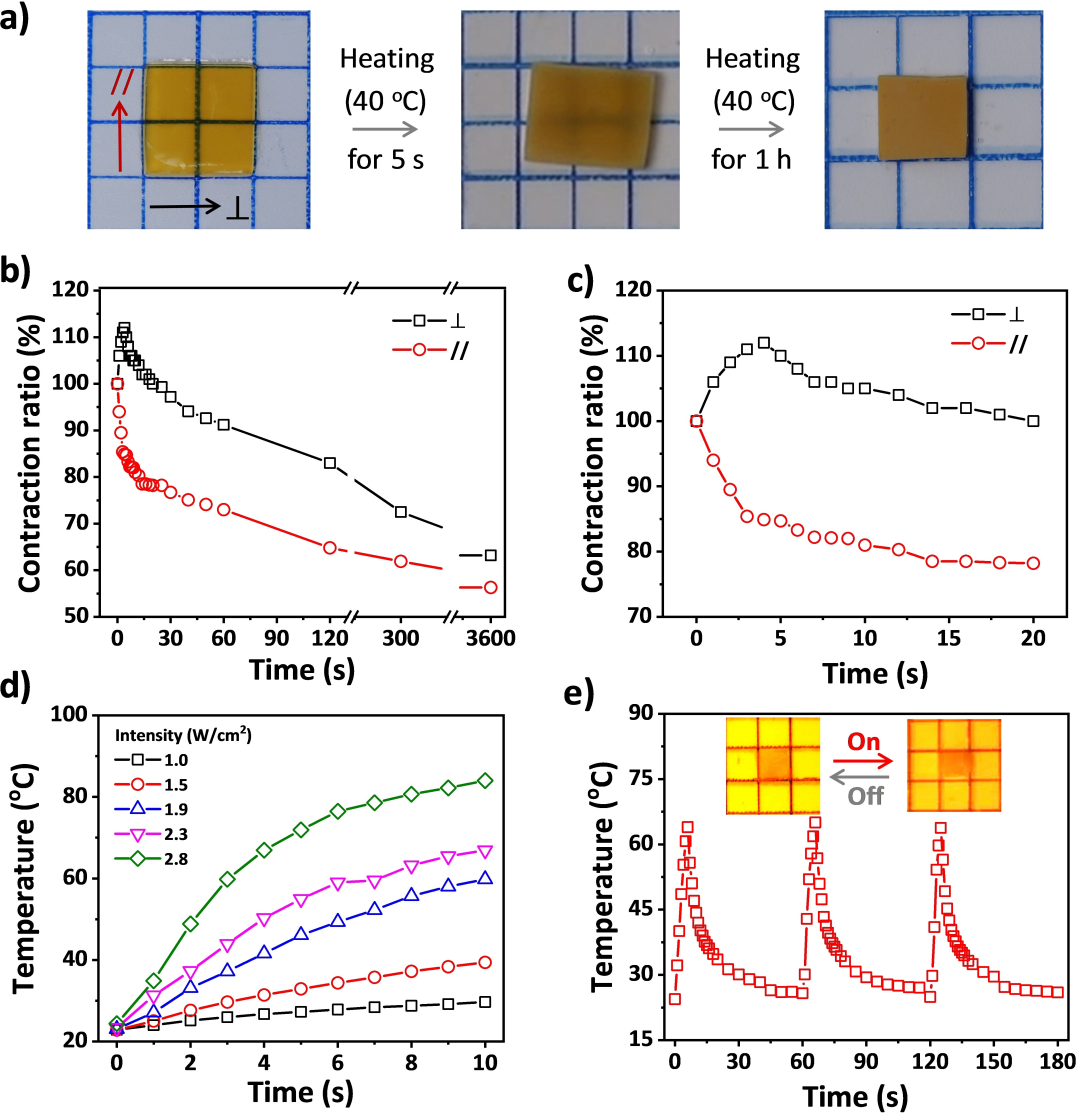
a) Photos showing the temperature‐mediated anisotropic deformation of the hydrogel. b, c) Variations of the dimensions of the hydrogel after transferring it from 25 to 40 °C in a water bath. The initial change of the dimensions is enlarged in (c). d) Variations of local temperature of the anisotropic gel under irradiation of 520 nm light with different power intensity. e) Variations of the local temperature of the gel under cyclic irradiation of 520 nm light with the intensity of 2.34 W cm^−2^. The insets present the anisotropic deformation of the gel under light irradiation.

The nanocomposite PNIPAm hydrogel also exhibits anisotropic response to stimulus (Figure 3). After being incubated in hot water (40 °C), the gel rapidly becomes opaque, expanding to 1.1 times the original length in the ⊥ direction and contracting to 0.85 in the // direction within 5 s (Figure 3a–c). The abnormal slight expansion in the ⊥ direction is ascribed to the increase in electrostatic permittivity of the media with more free water molecules arising from the rapid dehydration of PNIPAm above the lower critical solution temperature (LCST). After long‐term storage at 40 °C, the hydrogel gradually contracts its volume by shrinking the length by a factor of 0.63 and 0.56 in ⊥ and // direction, respectively. The long‐term behavior of the gel is similar to the common PNIPAm hydrogel, which contracts at high temperature by migration of water molecules out of the gel matrix.[^9a^, ^9b^, ^9c^] For the anisotropic hydrogel in this work, volume contraction of the gel completes after ≈1 h.

The interlayer γ‐Fe_2_O_3_ in MDSs endows the nanocomposite hydrogel with a wide absorption peak in the region of 400–600 nm (Figure S13) and thus high photothermal efficiency.^[21]^ Thereby, the hydrogel can be locally heated by irradiation under a green laser (520 nm). The rising speed and amplitude of the local temperature depend on the intensity and irradiation time of the green laser (Figure 3d). With higher intensity, the temperature increases rapidly until it reaches the saturation temperature. For example, the local temperature of the gel dramatically rises from 23 to 44 °C within 4 s under the laser irradiation with the intensity of 2.34 W cm^−2^. Cyclic laser irradiation results in dynamic modulation of the temperature, which increases when the laser is on and decreases when it is off (Figure 3e). The variations of the gel's dimensions under laser irradiation are similar to that upon heating (Figure S14). The photothermal effect of γ‐Fe_2_O_3_ nanoparticles allows thus for a repeated modulation of the gel by laser irradiation. Therefore, the anisotropic hydrogel has multiple responses to magnetic field, temperature, and light, providing versatility in realizing programmed morphing and motion of the gel. Another advantage of the hydrogel is its superparamagnetism at ambient temperature, which is important for magnetic imaging and biomedical applications to monitor the location of the gel. The anisotropic structure of the hydrogel has good stability after incubation in water for more than 6 months or after ten cycles of heating–cooling treatments (Figure S15).

**Figure 4:**
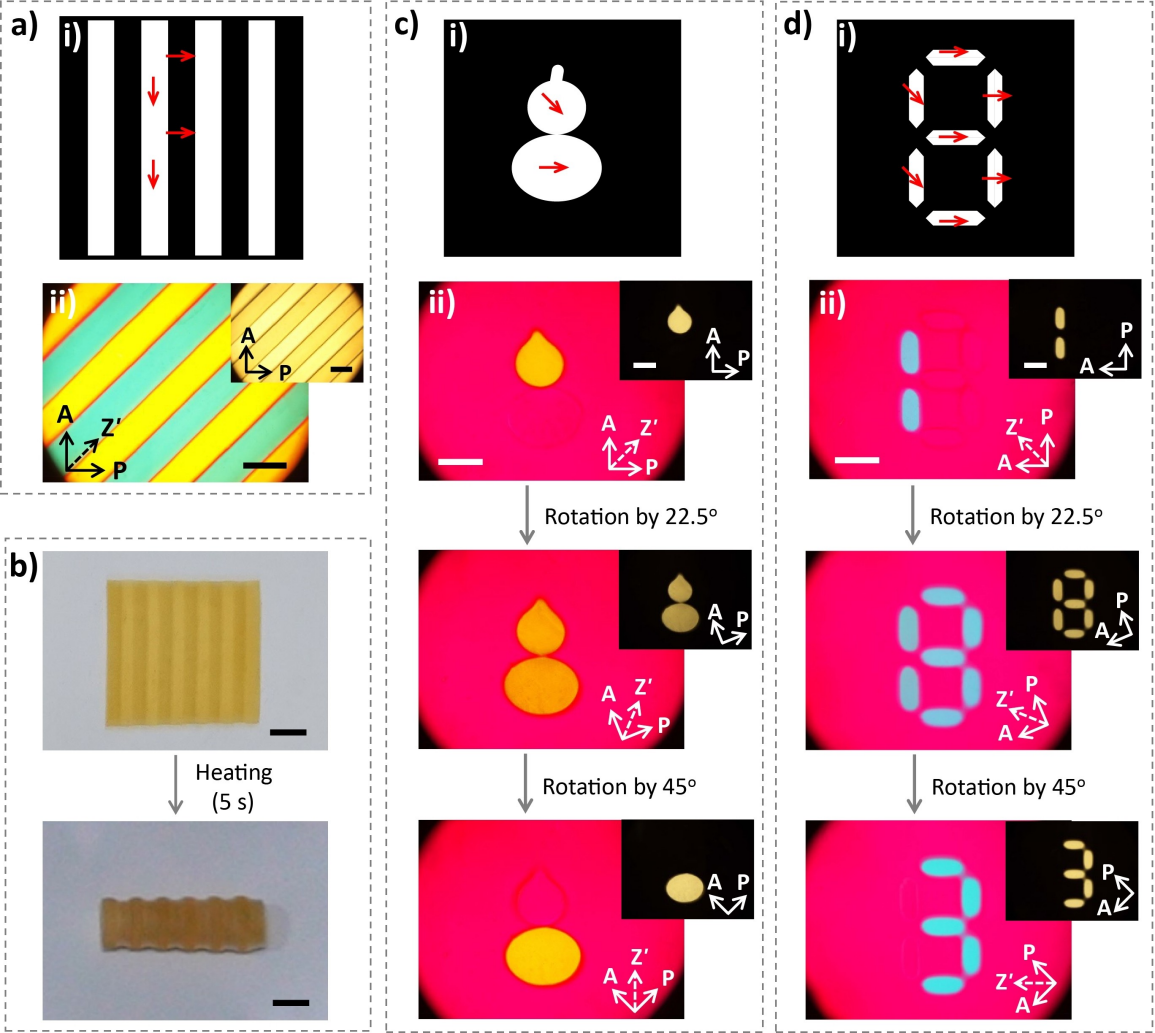
a) Schematic (i) and corresponding POM images (ii) of a stripe‐patterned anisotropic hydrogel. b) Photos of the stripe‐patterned anisotropic hydrogels before and after the shape changes upon incubation in a 40 °C warm water bath. The thickness of the sample is 0.6 mm. c,d) Schematic (i) and corresponding POM images (ii) of patterned hydrogels with different alignment of MDSs at specific regions by rotating the directions of analyzer, polarizer, and tint plate. The thickness of the samples is 0.4 mm. Scale bars in (a), (c), and (d) are 2 mm; scale bars in (b) are 5 mm.

To afford the gel with tailored morphing ability, it is highly desired yet challenging to form sophisticated anisotropic structures so as to control the distribution of internal stresses. Here, hydrogels with patterned anisotropic structures of MDSs are developed by multi‐step magnetic orientation and photolithographic polymerization. The stripe‐patterned hydrogel with orthogonal orientations of MDSs shows different birefringence colors when observed under POM (Figure 4a). This gel slightly bends along the stripe direction in water due to the different swelling ratios of the stripes with orthogonal orientations of MDSs (Figure 2d); the swelling mismatch builds up internal stresses along the stripe and leads to weak out‐of‐plane buckling deformation. The patterned gel deforms into cylinder shape when placed in 40 °C water, which drastically enhances the swelling/contraction mismatch, internal stresses, and deformation amplitude of the gel (Figure 4b).^[22]^ Different orientation of MDSs at specific regions can be produced by applying the rotating magnetic field and photo‐polymerizing with a mask. Repeated procedures enable fabrication of composite hydrogel with complex alignments of MDSs. For example, a pear‐like region is first polymerized with MDSs homotropically oriented in the southeastern direction, followed by creating an egg‐like region with MDSs homotropically oriented in the eastern direction. Finally, the other region is polymerized with MDSs homogeneously oriented along the planar direction. Different birefringent patterns can be observed under POM. An orange pear‐like pattern is observed when the gourd‐shape is placed along the analyzer direction of the POM. After anticlockwise rotating the analyzer, polarizer, and tint plate by 22.5°, a gourd‐like pattern with orange birefringence is observed. Another anticlockwise rotation by 22.5° results in an egg‐like pattern with strong orange birefringence (Figure 4c); other birefringent patterns observed after further rotations are presented in Figure S16a. Similarly, birefringent patterns of digital numbers can also be encoded in the anisotropic gel. The homotropic alignments of MDSs and corresponding birefringent patterns are shown in Figure 4d and Figure S16b. Different birefringent numbers are observed in the same hydrogel, which depend on the relative position of the oriented direction of MDSs and the polarizers of POM. We should note that the patterns of the gels developed by multi‐step polymerization are almost invisible under daylight (Figure S17). These properties of the patterned anisotropic hydrogels should be promising for camouflage and encryption of digital information. The resolution of the patterns in this work is ≈1 mm, which can be improved to tens of micrometers by using high‐resolution masks and increasing the photopolymerization rate.^[23]^

**Figure 5:**
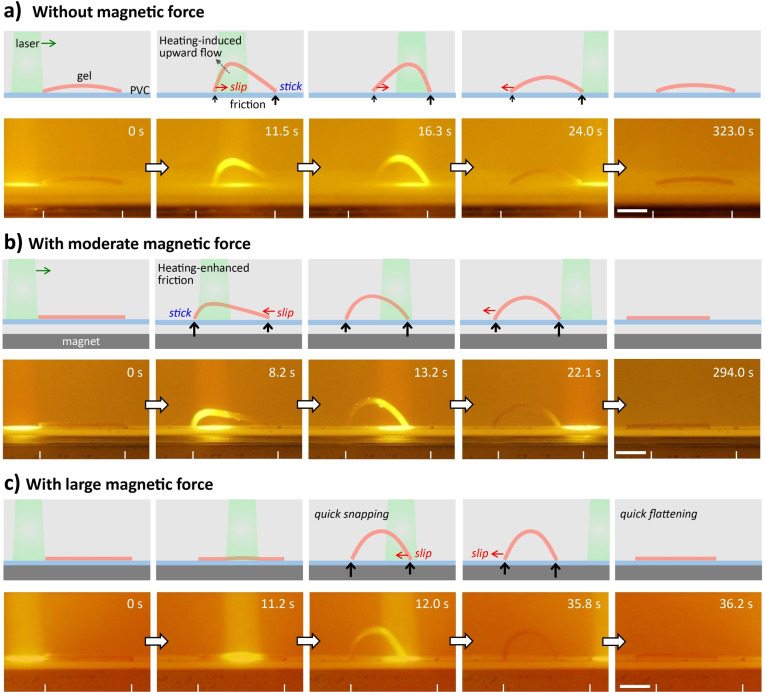
Snapshots showing the walking gait of the stripe‐patterned anisotropic hydrogel under a scanning laser beam from left to right without magnetic force (a), with moderate magnetic force (b) and large magnetic force (c). Schematics are presented above the snapshots to show the motions of the two “feet”. Gel dimensions, 15 mm×5 mm×0.6 mm; light intensity, 2.34 W cm^−2^; scanning speed, 1 mm s^−1^. Scale bar: 5 mm.

The photo‐responsiveness of the anisotropic hydrogel, moreover, enables local actuation for shape morphing and programmed locomotion. As a demonstration, the stripe‐patterned hydrogel film previously shown in Figure 4a, which has alternating alignments of MDSs parallel or perpendicular to the stripe direction, is placed on a hydrophobic polyvinyl chloride (PVC) substrate and actuated by repeated unidirectional scanning (from left to right) of a laser beam to achieve a walking motion. Due to the swelling mismatch between the stripes with different alignments of MDSs, the patterned hydrogel slightly bends in water at room temperature.[^9b^, ^24^] Localized irradiation dramatically increases the local temperature and improves the swelling/contraction mismatch, the internal stresses, and thus the bending amplitude of the stripe‐patterned hydrogel.^[9b]^ We initially expected the gel with a walking gait after cyclic directional scanning of the laser, which results in dynamic shape‐changing and friction modulation of the gel against the PVC substrate by the hydrophilic‐to‐hydrophobic transition of PNIPAm matrix, as revealed in a previous work.^[9b]^ However, the light‐steered motion of this gel is not so efficient (Figure 5a and Movie S1), because the density of the gel (1.012±0.003 g cm^−3^) is too close to water (0.997 g cm^−3^) at room temperature. When the laser beam scans from left to right, the hydrogel shows travelling bending with the left “foot” moving toward the middle, while the right “foot” remains static. The moving of the left “foot” in the same direction of the scanning laser results in a prolonged irradiation on the left part of the gel. The preferential moving of the left “foot” is probably because the local heating of the gel leads to upward flow of surrounding water and reduced friction of the left “foot” against the substrate. After the laser beam reaches the middle of the gel, the bending of the gel reaches the maximum amplitude. When the laser continuously moves right, the recovery of the left part of the gel results in a movement of the left “foot” to the left. The right “foot” shows little displacement because of the much shorter time of laser irradiation. The uncontrollable friction of the “feet” against the substrate leads to negligible displacement of the intact hydrogel after one cycle of laser scanning.

To enhance the friction force so as to favor the locomotion, a magnet is placed beneath the hydrogel with a certain distance to generate magnetic force and thus to reduce the influence of buoyant force of the gel. Simulation results indicate that the hydrogel experiences magnetic force, which decreases with increasing distance between gel and magnet (Figure S18). When the magnetic force is applied with a gel‐magnet distance of 2.7 mm, the gel shows a programmed walking motion under cyclic irradiation of a laser beam (Figure 5b and Movie S2). Due to the existing magnetic force, the slightly curved gel is now flattened. As the laser beam scans on the left part of the gel, the gel gradually deforms with the right “foot” moving left because the left “foot” with elevated temperature and enhanced hydrophobicity sticks on the hydrophobic substrate. As the laser moves to the right part of the gel, the left “foot” with decreased temperature moves left, and the right “foot” with elevated temperature sticks on the substrate. Such dynamic modulation of the friction force, together with the travelling bending deformation, leads to a high‐efficiency directional walking motion of the hydrogel.

When the magnetic force is further increased by decreasing the gel‐magnet distance to 0.3 mm, the light‐steered motion is different from the aforementioned situations (Figure 5c and Movie S3). The gel cannot deform initially under the scanning laser beam because the internal stress within the gel is not sufficiently high to overcome the magnetic force and the friction force. As the light scanning continues, the internal stress gradually increases, which is released by a sudden move of the right “foot” of the gel to the left (completed in ≈1 s). As a consequence, the gel deforms into an arch. After switching off laser irradiation, the recovery of the gel from the arch to flat shape also completes abruptly with the gel instantly falling onto the substrate, when the gel recovers its volume and the unbending force is larger than the friction force to trigger the sliding of the “feet” on the substrate. After the occurrence of sliding, the flattening process of the gel is accelerated by the magnetic force. The sudden buckling and flattening of the hydrogel should be associated with the stick‐slip transition under the competition between the internal stress and the friction force.

The distinct deformations and motions of the hydrogel manipulated by the magnetic field and photo actuation are related to the anisotropic structure and the multi‐response of the MDSs. This combination of different stimulus‐response properties of the hydrogels should facilitate the design and control of soft actuators and robots toward specific applications. Worth noting is that the multi‐gait motions demonstrated above are different from the reported examples in terms of the kinematics (Table S1). In the achievements reported in the literatures, two strategies are usually applied to convert cyclic bending/unbending or stretching/contraction deformations into directional walking motions, i.e. by using a rachet plate or creating asymmetric shape of the gel to generate asymmetric friction against the substrate.^[15]^ However, the hydrogel usually moves along predetermined direction, cannot move in the reverse direction or with a distinct gait. These issues have been addressed by cooperative manipulation of the magnetic field and spatiotemporal laser irradiation to afford the patterned gel with multi‐gait motions. A static magnetic field is applied to adjust the friction between the gel and the substrate, which changes the motion gait under dynamic light stimulation. The combined strategy should be informative for the design of soft actuators and robots.

## Conclusion

In summary, we have developed a series of anisotropic hydrogels by using a rotating magnetic field to orient MDSs followed by polymerization of the precursor to fix the ordered structures. The resultant PNIPAm nanocomposite hydrogels with highly ordered MDSs have anisotropic optical and mechanical properties, and exhibit anisotropic responses to temperature or light irradiation. These properties are rooted in the unique attributes of MDSs with a sandwich‐like structure, high aspect ratio, high charge density, and responsiveness to light and magnetic field. Hydrogels with sophisticated anisotropic structures of MDSs are further fabricated by multi‐step magnetic orientation and photolithographic polymerization, which show different birefringent patterns and programmed deformations upon heating or light irradiation. Distinct motions are realized in the patterned hydrogel under spatiotemporal light irradiation with a static magnet to regulate the friction force between the gel and the substrate. The magneto‐orientation‐assisted fabrication of anisotropic hydrogels and the cooperative manipulation strategy for controllable motions should be informative for the design of high‐performance hydrogel devices. These hydrogels with controllable anisotropic structures, multiple stimuli‐responsiveness, programmable deformations and motions should find applications in biomedical and engineering fields as biomedical devices, soft actuators/robots, etc.

## Conflict of interest

The authors declare no conflict of interest.

1

## Supporting information

As a service to our authors and readers, this journal provides supporting information supplied by the authors. Such materials are peer reviewed and may be re‐organized for online delivery, but are not copy‐edited or typeset. Technical support issues arising from supporting information (other than missing files) should be addressed to the authors.

Supporting Information

Supporting Information

Supporting Information

Supporting Information

## Data Availability

The data that support the findings of this study are available from the corresponding authors upon reasonable request.

## References

[1a] U. G. K. Wegst , H. Bai , E. Saiz , A. P. Tomsia , R. O. Ritchie , Nat. Mater. 2015, 14, 23; 25344782 10.1038/nmat4089

[1b] M. Eder , S. Amini , P. Fratzl , Science 2018, 362, 543; 30385570 10.1126/science.aat8297

[1c] D. Jiao , Q. L. Zhu , C. Y. Li , Q. Zheng , Z. L. Wu , Acc. Chem. Res. 2022, 55, 1533. 35413187 10.1021/acs.accounts.2c00046

[2a] K. Wang , J. Ren , S. Yang , H. Wang , Adv. Mater. Technol. 2021, 6, 2100158;

[2b] Y. S. Zhang , A. Khademhosseini , Science 2017, 356, eaaf3627; 28473537 10.1126/science.aaf3627 PMC5841082

[2c] Y. Zhao , C. Xuan , X. Qian , Y. Alsaid , M. Hua , L. Jin , X. He , Sci. Robot. 2019, 4, eaax7112. 33137784 10.1126/scirobotics.aax7112

[3a] W. Wang , Y. Zhang , W. Liu , Prog. Polym. Sci. 2017, 71, 1;

[3b] X. Zhao , X. Chen , H. Yuk , S. Lin , X. Liu , G. Parada , Chem. Rev. 2021, 121, 4309; 33844906 10.1021/acs.chemrev.0c01088 PMC9217625

[3c] Y. J. Wang , X. N. Zhang , Y. Song , Y. Zhao , L. Chen , F. Su , L. Li , Z. L. Wu , Q. Zheng , Chem. Mater. 2019, 31, 1430;

[3d] C. N. Zhu , T. Bai , H. Wang , J. Ling , F. Huang , Z. L. Wu , Adv. Mater. 2021, 33, 2102023; 10.1002/adma.202102023 34081366

[3e] Z. Jiang , M. L. Tan , M. Taheri , Q. Yan , T. Tsuzuki , M. G. Gardiner , B. Diggle , L. A. Connal , Angew. Chem. Int. Ed. 2020, 59, 7049; 10.1002/anie.201916058 32167650

[4a] M. J. Wakelam , Biochem. J. 1985, 228, 1; 3890835 10.1042/bj2280001 PMC1144947

[4b] S. Jana , S. K. L. Levengood , M. Zhang , Adv. Mater. 2016, 28, 10588; 27865007 10.1002/adma.201600240 PMC5253134

[4c] H. N. Kim , A. Jiao , N. S. Hwang , M. S. Kim , D. H. Kang , D. H. Kim , K. Y. Suh , Adv. Drug Delivery Rev. 2013, 65, 536; 10.1016/j.addr.2012.07.014 PMC5444877 22921841

[4d] J. G. Barber , A. M. Handorf , T. J. Allee , W. J. Li , Tissue Eng. Part A 2013, 19, 1265. 21895485 10.1089/ten.tea.2010.0538

[5a] M. T. I. Mredha , I. Jeon , Prog. Mater. Sci. 2022, 124, 100870;

[5b] K. Sano , Y. Ishida , T. Aida , Angew. Chem. Int. Ed. 2018, 57, 2532; 10.1002/anie.201708196 29034553

[5c] N. Peng , D. Huang , C. Gong , Y. Wang , J. Zhou , C. Chang , ACS Nano 2020, 14, 16169; 10.1021/acsnano.0c08906 33314921

[5d] Z. Zhao , R. Fang , Q. Rong , M. Liu , Adv. Mater. 2017, 29, 1703045. 10.1002/adma.201703045 29059482

[6a] W. Yang , S. Yamamoto , K. Sueyoshi , T. Inadomi , R. Kato , N. Miyamoto , Angew. Chem. Int. Ed. 2021, 60, 8466; 10.1002/anie.202015982 33480099

[6b] T. Inadomi , S. Ikeda , Y. Okumura , H. Hirotsugu , N. Miyamoto , Macromol. Rapid Commun. 2014, 35, 1741. 10.1002/marc.201400333 25228493

[7a] S. Choi , J. Kim , J. Mater. Chem. B 2015, 3, 1479; 32262420 10.1039/c4tb01852d

[7b] S. H. Kim , S. K. Im , S. J. Oh , S. Jeong , E. S. Yoon , C. J. Lee , N. Choi , E. M. Hur , Nat. Commun. 2017, 8, 14346; 28146148 10.1038/ncomms14346 PMC5296669

[7c] P. Lin , T. Zhang , X. Wang , B. Yu , F. Zhou , Small 2016, 12, 4386. 27376708 10.1002/smll.201601893

[8a] B. D. Wall , S. R. Diegelmann , S. Zhang , T. J. Dawidczyk , W. L. Wilson , H. E. Katz , H. Q. Mao , J. D. Tovar , Adv. Mater. 2011, 23, 5009; 22180891 10.1002/adma.201102963

[8b] N. Miyamoto , M. Shintate , S. Ikeda , Y. Hoshida , Y. Yamauchi , R. Motokawa , M. Annaka , Chem. Commun. 2013, 49, 1082; 10.1039/c2cc36654a 23283288

[8c] Z. Zhu , Y. Li , H. Xu , X. Peng , Y. N. Chen , C. Shang , Q. Zhang , J. Liu , H. Wang , ACS Appl. Mater. Interfaces 2016, 8, 15637; 27254730 10.1021/acsami.6b04325

[8d] K. Shikinaka , K. Kaneda , S. Mori , T. Maki , H. Masunaga , Y. Osada , K. Shigehara , Small 2014, 10, 1813. 24573908 10.1002/smll.201303360

[9a] Q. L. Zhu , C. Du , Y. Dai , M. Daab , M. Matejdes , J. Breu , W. Hong , Q. Zheng , Z. L. Wu , Nat. Commun. 2020, 11, 5166; 33056999 10.1038/s41467-020-18801-1 PMC7560679

[9b] Q. L. Zhu , C. F. Dai , D. Wagner , M. Daab , W. Hong , J. Breu , Q. Zheng , Z. L. Wu , Adv. Mater. 2020, 32, 2005567; 10.1002/adma.202005567 33079426

[9c] Q. L. Zhu , C. F. Dai , D. Wagner , O. Khoruzhenko , W. Hong , J. Breu , Q. Zheng , Z. L. Wu , Adv. Sci. 2021, 8, 2102353; 10.1002/advs.202102353 PMC8693068 34705341

[9d] Y. Guo , Y. Chen , E. Wang , M. Cakmak , ACS Appl. Mater. Interfaces 2017, 9, 919; 27982568 10.1021/acsami.6b13207

[9e] M. Yang , Y. Xu , X. Zhang , H. K. Bisoyi , P. Xue , Y. Yang , X. Yang , C. Valenzuela , Y. Chen , L. Wang , W. Feng , Q. Li , Adv. Funct. Mater. 2022, 32, 2201884;

[9f] T. Inadomi , K. Urayama , N. Miyamoto , ACS Appl. Polym. Mater. 2022, 10.1021/acsapm.2c00103;

[9g] P. Xue , H. K. Bisoyi , Y. Chen , H. Zeng , J. Yang , X. Yang , P. Lv , X. Zhang , A. Priimagi , L. Wang , X. Xu , Q. Li , Angew. Chem. Int. Ed. 2021, 60, 3390; 10.1002/anie.202014533 33259120

[10a] W. Shi , J. Huang , R. Fang , M. Liu , ACS Appl. Mater. Interfaces 2020, 12, 5177; 31916743 10.1021/acsami.9b16770

[10b] K. Li , J. Xu , P. Li , Y. Fan , Composites Part B 2022, 228, 109401.

[11a] J. Kim , S. E. Chung , S. E. Choi , H. Lee , J. Kim , S. Kwon , Nat. Mater. 2011, 10, 747; 21822261 10.1038/nmat3090

[11b] K. Hu , J. Sun , Z. Guo , P. Wang , Q. Chen , M. Ma , N. Gu , Adv. Mater. 2015, 27, 2507; 25753892 10.1002/adma.201405757

[11c] S. R. Goudu , I. C. Yasa , X. Hu , H. Ceylan , W. Hu , M. Sitti , Adv. Funct. Mater. 2020, 30, 2004975;

[11d] C. Li , G. C. Lau , H. Yuan , A. Aggarwal , V. L. Dominguez , S. Liu , H. Sai , L. C. Palmer , N. A. Sather , T. J. Pearson , D. E. Freedman , P. K. Amiri , M. O. de la Cruz , S. I. Stupp , Sci. Robot. 2020, 5, eabb9822. 33298516 10.1126/scirobotics.abb9822

[12a] L. Wu , M. Ohtani , M. Takata , A. Saeki , S. Seki , Y. Ishida , T. Aida , ACS Nano 2014, 8, 4640; 24738828 10.1021/nn5003908

[12b] T. Lan , B. Ding , Z. Huang , F. Bian , Y. Pan , H. M. Cheng , B. Liu , J. Am. Chem. Soc. 2021, 143, 12886; 34369770 10.1021/jacs.1c07481

[12c] S. Yook , S. Shams Es-Haghi , A. Yildirim , Z. Mutlu , M. Cakmak , Soft Matter 2019, 15, 9733; 31742299 10.1039/c9sm01789e

[12d] F. Lin , Z. Zhu , X. Zhou , W. Qiu , C. Niu , J. Hu , K. Dahal , Y. Wang , Z. Zhao , Z. Ren , D. Litvinov , Z. Liu , Z. M. Wang , J. Bao , Adv. Mater. 2017, 29, 1604453. 10.1002/adma.201604453 27862419

[13a] R. M. Erb , J. S. Sander , R. Grisch , A. R. Studart , Nat. Commun. 2013, 4, 1712; 23591879 10.1038/ncomms2666

[13b] R. M. Erb , R. Libanori , N. Rothfuchs , A. R. Studart , Science 2012, 335, 199; 22246772 10.1126/science.1210822

[13c] H. Le Ferrand , F. Bouville , T. P. Niebel , A. R. Studart , Nat. Mater. 2015, 14, 1172; 26390326 10.1038/nmat4419

[13d] E. Poloni , A. Rafsanjani , V. Place , D. Ferretti , A. R. Studart , Adv. Mater. 2022, 34, 2104874. 10.1002/adma.202104874 34632656

[14a] M. Liu , Y. Ishida , Y. Ebina , T. Sasaki , T. Hikima , M. Takata , T. Aida , Nature 2015, 517, 68; 25557713 10.1038/nature14060

[14b] Z. Sun , Y. Yamauchi , F. Araoka , Y. S. Kim , J. Bergueiro , Y. Ishida , Y. Ebina , T. Sasaki , T. Hikima , T. Aida , Angew. Chem. Int. Ed. 2018, 57, 15772; 10.1002/anie.201810052 30315618

[14c] K. Sano , Y. O. Arazoe , Y. Ishida , Y. Ebina , M. Osada , T. Sasaki , T. Hikima , T. Aida , Angew. Chem. Int. Ed. 2018, 57, 12508; 10.1002/anie.201807240 30073724

[14d] Y. S. Kim , M. Liu , Y. Ishida , Y. Ebina , M. Osada , T. Sasaki , T. Hikima , M. Takata , T. Aida , Nat. Mater. 2015, 14, 1002. 26259107 10.1038/nmat4363

[15a] H. Shahsavan , A. Aghakhani , H. Zeng , Y. Guo , Z. S. Davidson , A. Priimagi , M. Sitti , Proc. Natl. Acad. Sci. USA 2020, 117, 5125; 32094173 10.1073/pnas.1917952117 PMC7071923

[15b] Z.-C. Jiang , Y.-Y. Xiao , X. Tong , Y. Zhao , Angew. Chem. Int. Ed. 2019, 58, 5332; 10.1002/anie.201900470 30816599

[16a] O. Khoruzhenko , D. R. Wagner , S. Mangelsen , M. Dulle , S. Förster , S. Rosenfeldt , V. Dudko , K. Ottermann , G. Papastavrou , W. Bensch , J. Breu , J. Mater. Chem. C 2021, 9, 12732;

[16b] K. Ament , D. R. Wagner , F. E. Meij , F. E. Wagner , J. Breu , Z. Anorg. Allg. Chem. 2020, 646, 1110.

[17a] S. Rosenfeldt , M. Stöter , M. Schlenk , T. Martin , R. Q. Albuquerque , S. Förster , J. Breu , Langmuir 2016, 32, 10582; 27648496 10.1021/acs.langmuir.6b02206

[17b] M. Daab , S. Rosenfeldt , H. Kalo , M. Stöter , B. Bojer , R. Siegel , S. Förster , J. Senker , J. Breu , Langmuir 2017, 33, 4816; 28452487 10.1021/acs.langmuir.7b01008

[17c] M. Stöter , S. Godrich , P. Peicht , S. Rosenfeldt , H. Thurn , J. W. Neubauer , M. Seuss , P. Lindner , H. Kalo , M. Moller , A. Fery , S. Porster , G. Papastavrou , J. Breu , Angew. Chem. Int. Ed. 2016, 55, 7398; 10.1002/anie.201601611 27140654

[18] L. Qiao , C. Du , J. P. Gong , Z. L. Wu , Q. Zheng , Adv. Mater. Technol. 2019, 4, 1900665.

[19a] R. M. Erb , J. Segmehl , M. Schaffner , A. R. Studart , Soft Matter 2013, 9, 498;

[19b] H. Le Ferrand , A. F. Arrieta , Soft Matter 2022, 18, 1054. 35022646 10.1039/d1sm01423d

[20] H. Qin , T. Zhang , N. Li , H. Cong , S. Yu , Nat. Commun. 2019, 10, 2202. 31101823 10.1038/s41467-019-10243-8 PMC6525195

[21a] K. Li , H. Nejadnik , H. E. Daldrup-Link , Drug Discovery Today 2017, 22, 1421; 28454771 10.1016/j.drudis.2017.04.008 PMC5610947

[21b] M. Saeed , W. Ren , A. Wu , Biomater. Sci. 2018, 6, 708; 29363682 10.1039/c7bm00999b

[21c] P. Martinkova , M. Brtnicky , J. Kynicky , M. Pohanka , Adv. Healthcare Mater. 2018, 7, 1700932. 10.1002/adhm.201700932 29205944

[22] H. Aharoni , Y. Xia , X. Zhang , R. D. Kamien , S. Yang , Proc. Natl. Acad. Sci. USA 2018, 115, 7206. 29929963 10.1073/pnas.1804702115 PMC6048487

[23a] S.-J. Jeon , A. W. Hauser , R. C. Hayward , Acc. Chem. Res. 2017, 50, 161; 28181798 10.1021/acs.accounts.6b00570

[23b] A. Cangialosi , C. Yoon , J. Liu , Q. Huang , J. Guo , T. D. Nguyen , D. H. Gracias , R. Schulman , Science 2017, 357, 1126; 28912239 10.1126/science.aan3925

[23c] F. Huang , M. Chen , Z. Zhou , R. Duan , F. Xia , I. Willner , Nat. Commun. 2021, 12, 2364. 33888708 10.1038/s41467-021-22645-8 PMC8062675

[24a] Z. L. Wu , M. Moshe , J. Greener , H. Therien-Aubin , Z. Nie , E. Sharon , E. Kumacheva , Nat. Commun. 2013, 4, 1586; 23481394 10.1038/ncomms2549

[24b] C. N. Zhu , C. Y. Li , H. Wang , W. Hong , F. Huang , Q. Zheng , Z. L. Wu , Adv. Mater. 2021, 33, 2008057; 10.1002/adma.202008057 33788313

[24c] C. Y. Li , S. Y. Zheng , X. P. Hao , W. Hong , Q. Zheng , Z. L. Wu , Sci. Adv. 2022, 8, eabm9608. 35417235 10.1126/sciadv.abm9608 PMC9007498

