# Supplementary material for: Magneto‐Orientation of Magnetic Double Stacks for Patterned Anisotropic Hydrogels with Multiple Responses and Modulable Motions
Source: Angew Chem Int Ed Engl. 2022 Jul 14;61(35):e202207272. doi: 10.1002/anie.202207272 (PMC9541020; doi:10.1002/anie.202207272)
Supplement: Supplementary file 1 — Supporting Information [file ANIE-61-0-s001.pdf]

Angewandte Chemie
Eine Zeitschrift der Gesellschaft Deutscher Chemiker

Supporting Information

Magneto-Orientation of Magnetic Double Stacks for Patterned Anisotropic Hydrogels with Multiple Responses and Modulable Motions

C. F. Dai, O. Khoruzhenko, C. Zhang, Q. L. Zhu, D. Jiao, M. Du, J. Breu, P. Zhao*, Q. Zheng*, Z. L. Wu**

Experimental Section

Materials

N-isopropylacrylamide (NIPAm) was used as received from Tokyo Chemical Industry Co., Ltd. *N*,*N′*-methylenebis(acrylamide) (MBAA) was purchased from Aladdin Chemistry Co., Ltd. Lithium phenyl-2,4,6-trimethylbenzoylphosphinate (LAP) was synthesized according to the reported process.[S1] MDS was synthesized by adapting the reported procedure.[S2] The content of γ-Fe_2O_3 was fixed at 16.6 wt%. Aqueous suspensions of MDS (1 wt%) were prepared by adding prescribed amount of MDS power to water. The mixture was oscillated with a speed of 120 r.p.m. for 24 h at room temperature, which led to repulsive osmotic delamination into a ferronematic suspension consisting of singular MDSs with a sandwich-like structure. Millipore deionized water was used in all the experiments.

Synthesis of magnetic anisotropic hydrogels

The precursor suspension was prepared by dissolving a prescribed amount of NIPAm (1 mol/L), MBAA (3 mol%, relative to NIPAm), and LAP (6 mol%, relative to NIPAm) in the homogeneous suspension of MDS (1 wt%). After injecting the aqueous precursor suspension into the reaction cell consisting of a pair of parallel glass substrates with silicon spacer of a specific thickness, a static or rotating magnetic field was applied to direct the alignment of MDSs. After the magnetic orientation for a short while, the reaction cell was immediately exposed to UV light for 10 s to initiate the polymerization and crosslinking to obtain the anisotropic hydrogel.

The isotropic hydrogel containing randomly dispersed MDSs was prepared according to a similar protocol. After injection of the precursor, the reaction cell was oscillated with a speed of 60 rpm for 2 h at an elevated temperature to accelerate the structural relaxation of the shear-induced alignment of MDSs. Then, the reaction cell was cooled down to room temperature and placed under UV light irradiation for 10 s to trigger the polymerization. The obtained hydrogel was incubated in water to achieve the equilibrium state.

Patterned anisotropic hydrogels with complex ordered structures were fabricated by a multi-step magnetic orientation of MDSs and photolithographic polymerization. After the magnetic orientation of MDSs in the precursor suspension, the reaction cell was exposed to UV light for 10 s through a photo mask. After rotating the reaction cell to a certain angle, the rotating magnetic field was applied for 20 s, and the sample covered with another photo mask (or without photo mask) was exposed to

UV light for 10 s, resulting in polymerization of the precursor at specific regions. The process was repeated until the whole precursor suspension was polymerized to fix the ordered structures of the MDSs. The obtained patterned hydrogel was incubated into a large amount of water for several days to remove the residuals and achieve the equilibrium state.

Locomotion of the stripe-patterned hydrogels

Motions of the stripe-patterned hydrogel were realized under cyclic scanning of a laser beam (wavelength: 520 nm; intensity: 2.34 W/cm^2; spot diameter: 7 mm) from left to right of the rectangular hydrogel film (dimensions: 15 mm × 5 mm × 0.6 mm) placed on the polyvinyl chloride (PVC) substrate. The magnetic force was applied and adjusted by placing a N52 magnet (dimensions: 10 cm × 1 cm × 0.5 cm) under the PVC substrate with a specific distance. The locomotion was recorded by a digital camera with a cut-off filter (550–1100 nm) to filter out the strong green light.

Characterizations

The surface topography of MDSs was determined by atomic force microscopic (AFM) measurements. The image was acquired with a Dimension Icon (Bruker Nano Inc.) in PeakForce tapping mode in air. Magnetic hysteresis loops of γ-Fe_2O_3 nanoparticles, MDS powders, MDS suspension, and nanocomposite hydrogel were measured at room temperature by a Superconducting Quantum Interference Device (MPMS-XL-5) with the maximum applied magnetic field of 20000 Oe (Figure S19). The distribution of magnetic force of the nanocomposite hydrogel atop the elongated magnet was obtained by COMSOL simulation.

Absorption spectra were obtained by a UV-1800 spectrometer (Shimadzu Corp., Japan) at room temperature. The anisotropic gel was kept in a quartz cuvette with an optical path of 1 mm for measurement. To monitor the photothermal effect of the hydrogel, the localized temperature under irradiation of green light (520 nm) was measured by an infrared imager (Fotric 285).

The birefringent photos of MDS suspensions (1 wt %) were taken under a pair of polarizing films. The anisotropic hydrogels were observed under a POM (LV100N POL, Nikon) with and without a 530 nm tint plate. The gels with thickness of 0.5 mm were cut into strips with a width of ~0.5 mm for the cross-section observations. SAXS measurements were conducted on Xeuss SAXS system (Xenocs SA) with X-ray wavelength of 0.154 nm and beam spot of 172 × 172 μm^2. The distance between the sample and the detector was 1371 mm. The orientation degree (π) of MDSs in the hydrogel was calculated according to the equation of $\pi = (180–H)/180$, where H is the half width

of the peak of the azimuthal plot from the selected equatorial reflection.

The variations in // and ⊥ direction of the hydrogel prepared with rotating magnetic field are calculated as $S\,(//) = L_1/L_0$, and $S\,(\perp) = W_1/W_0$, respectively, in which L and W are the dimension in // and ⊥ directions. The subscript numbers 1 and 0 correspond to the deformed state and the original equilibrated state, respectively. The dimensions of hydrogels were analyzed from the snapshots of a movie that recorded the fast shape deformation of the gel after being transferred from a 25 °C water bath into a 40 °C water bath or being directly irradiated under 520 nm green light (intensity, 2.34 W/cm^2) at room temperature.

The mechanical properties of anisotropic and isotropic hydrogels containing MDSs were measured at room temperature using a tensile tester (Instron 3343). The anisotropic gel was cut into dumbbell-shaped samples, with a gauge length of 12 mm and a width of 2 mm, along the // or ⊥ direction. The isotropic hydrogel (without long-range orientation of MDSs) was also tested for comparison. Tensile tests were performed at a stretching rate of 100 mm/min. Young's modulus (E) (calculated with a strain below 7%), tensile breaking stress (σ_b), and breaking strain (ε_b) were obtained from three parallel measurements.

References

[S1] a) E. Majima, W. Schnabel, W. Weber, *Macromol. Chem. Phys.* **1991**, *192*, 2307; b) B. D. Fairbanks, M. P. Schwartz, C. N. Bowman, K. S. Anseth, *Biomaterials* **2009**, *30*, 6702.

[S2] O. Khoruzhenko, D. R. Wagner, S. Mangelsen, M. Dulle, S. Förster, S. Rosenfeldt, V. Dudko, K. Ottermann, G. Papastavrou, W. Bensch, J. Breu, *J. Mater. Chem. C* **2021**, *9*, 12732.

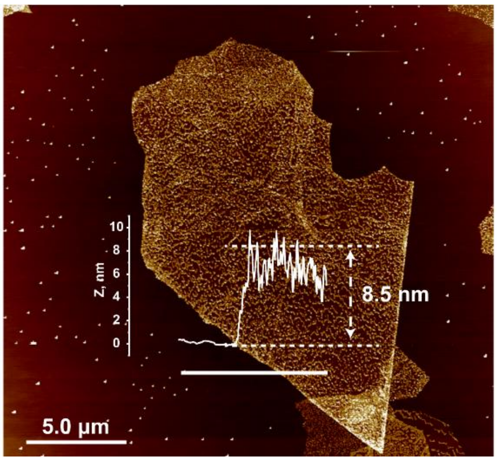

Figure S1. Topographical atomic force microscope (AFM) image and the height-length curves of an MDS.

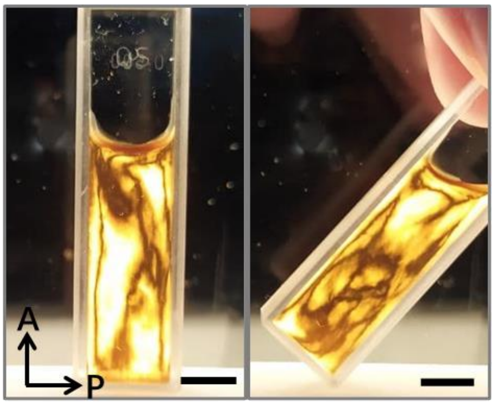

Figure S2. Photos of a ferronematic aqueous suspension with 1 wt% of MDS under crossed polarizing films. A: analyzer; P: polarizer. Scale bar: 5 mm.

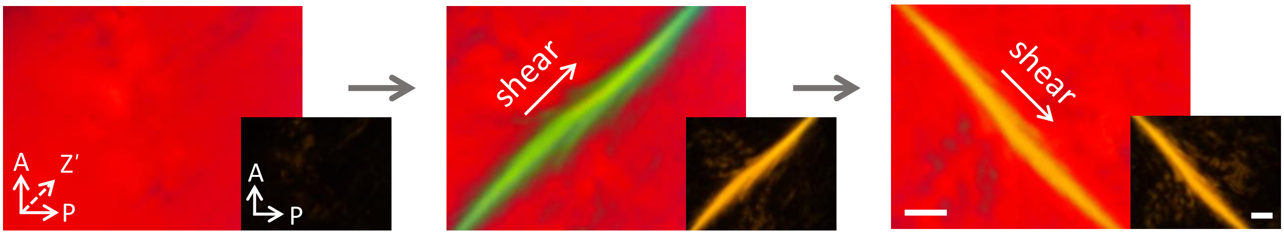

Figure S3. Mechanical shear-induced orientation of MDSs in suspension. Aqueous suspension with 1 wt% of MDS was dripped on a glass substrate, and a mechanical shear was applied to the suspension by using a syringe needle, which resulted in local orientation of MDSs along the shearing direction and strong birefringence under POM. A: analyzer; P: polarizer; Z′: slow axis of the 530 nm tint plate. Scale bar: 1 mm.

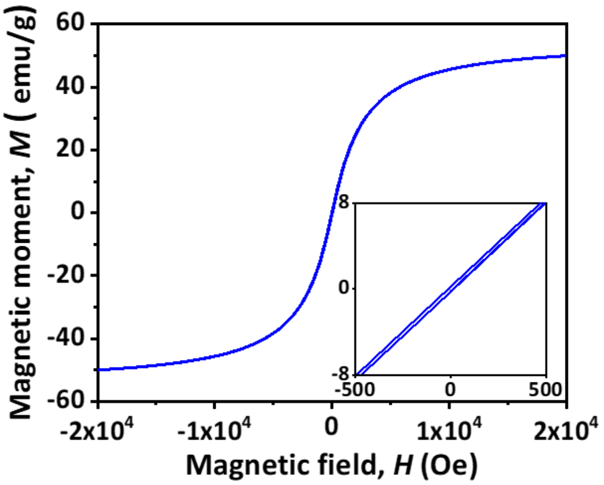

Figure S4. Magnetic hysteresis loop of γ-Fe_2O_3 nanoparticles. The diameter of γ-Fe_2O_3 nanoparticles is 5.5 ± 1.1 nm.

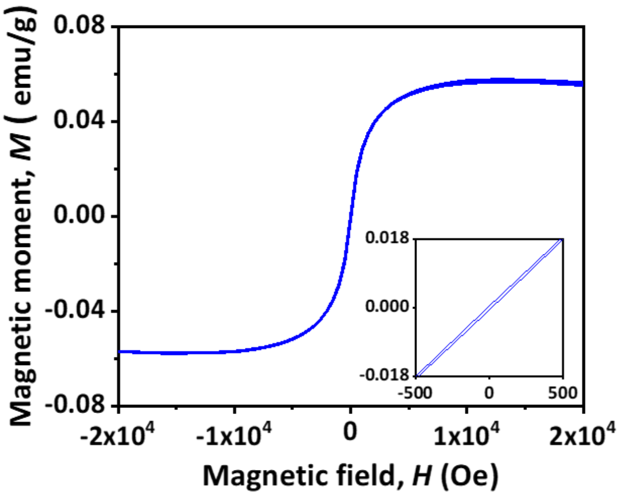

Figure S5. Magnetic hysteresis loop of aqueous suspension with 1 wt% of MDS.

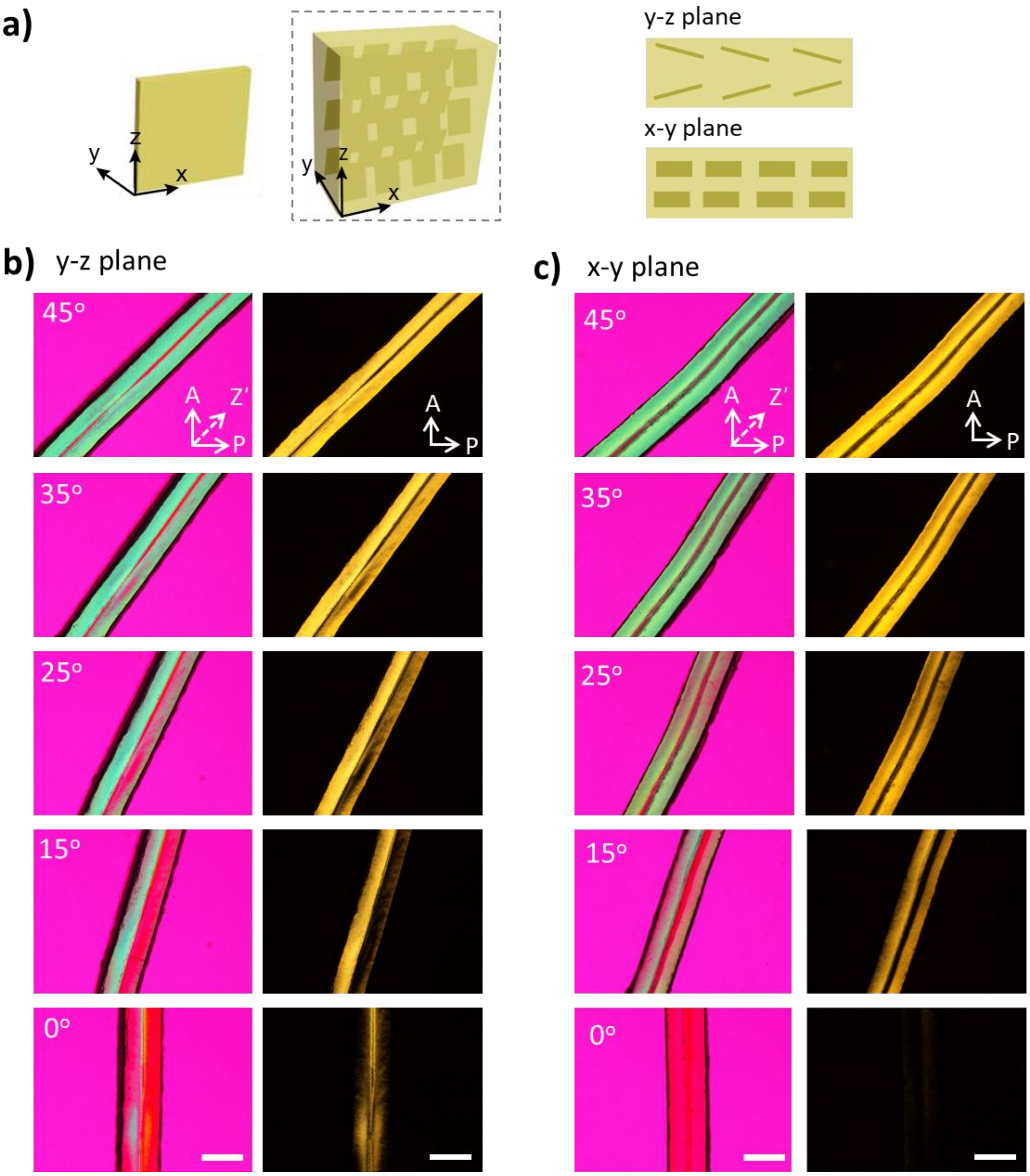

Figure S6. (a) Schematic showing the alignments of MDSs in the hydrogel prepared in the absence of magnetic fields. (b,c) POM images of the hydrogel observed from different directions: (b) y-z plane; (c) x-y plane. The sample was gradually rotated and then observed under POM with and without tint plate. The angle between the hydrogel and the analyzer is given in the POM image. Scale bar: 1 mm.

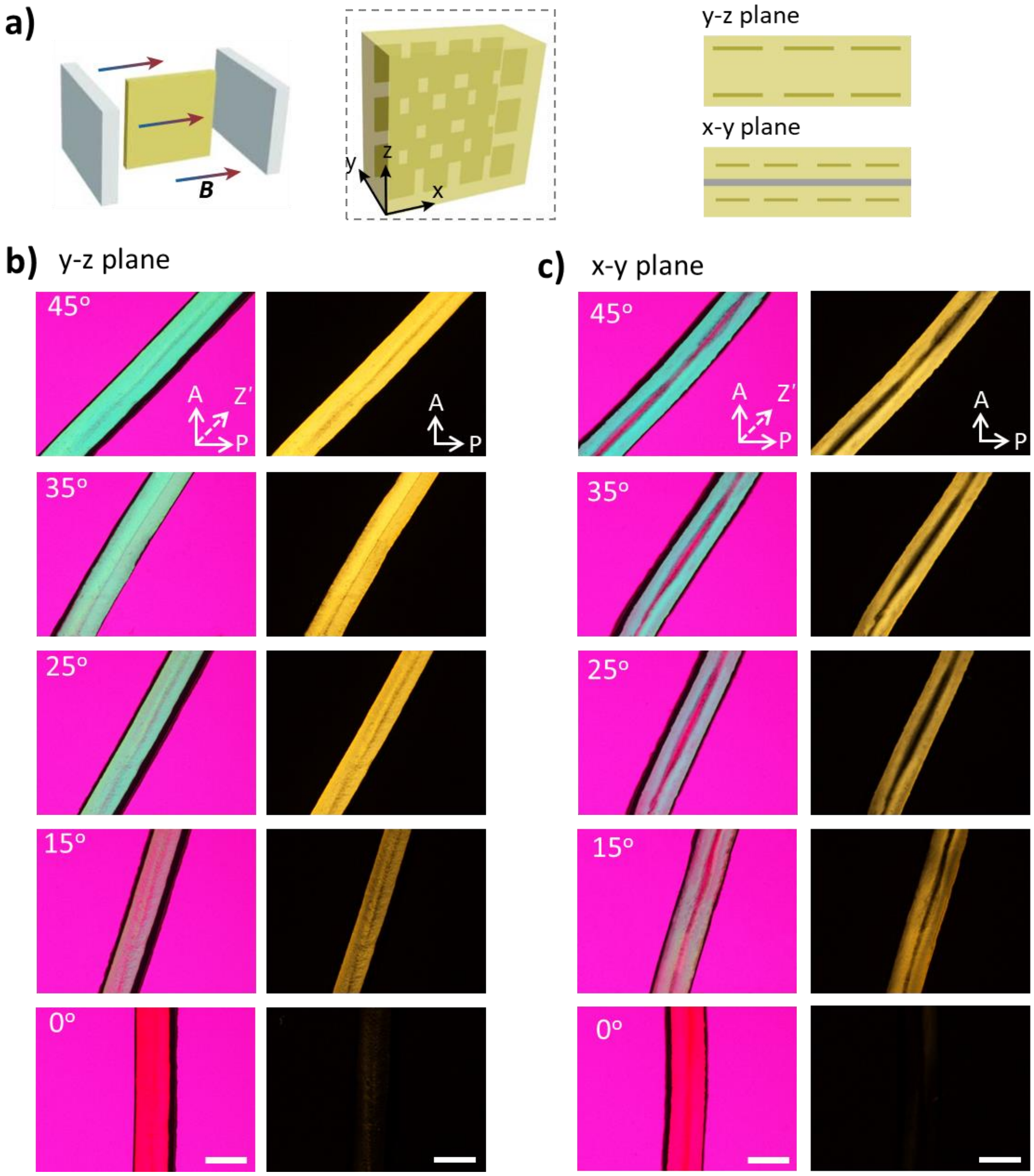

Figure S7. (a) Schematic showing the alignments of MDSs in the hydrogel prepared with static magnetic fields applied. (b,c) POM images of the hydrogel observed from different directions: (b) y-z plane; (c) x-y plane. The sample was gradually rotated and then observed under POM with and without tint plate. The angle between the hydrogel and the analyzer is given in the POM image. Scale bar: 1 mm.

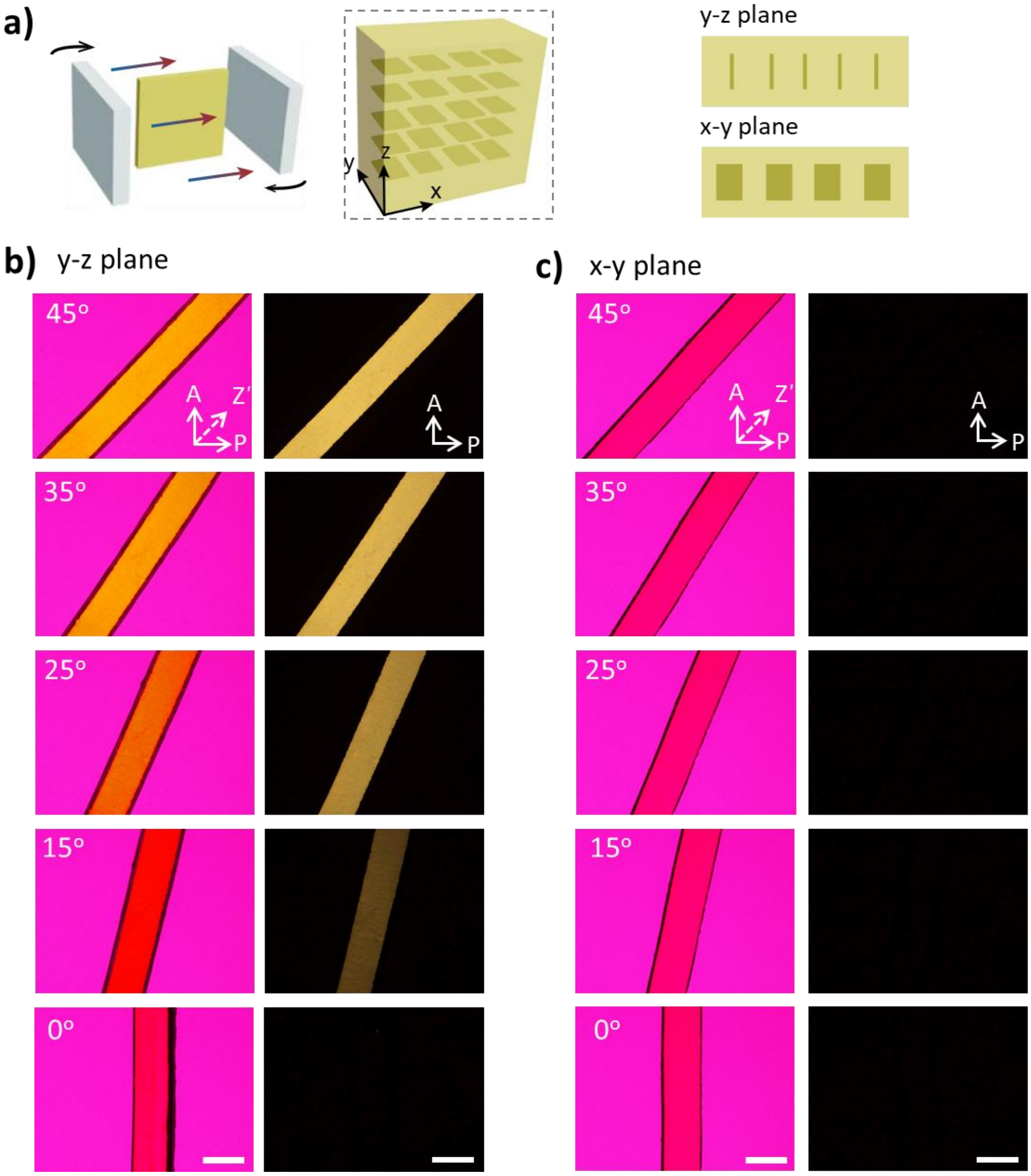

Figure S8. (a) Schematic showing the alignments of MDSs in the hydrogel prepared with rotating magnetic fields. (b,c) POM images of the hydrogel observed from different directions: (b) y-z plane; (c) x-y plane. The sample was gradually rotated and then observed under POM with and without tint plate. The angle between the hydrogel and the analyzer is given in the POM image. Scale bar: 1 mm.

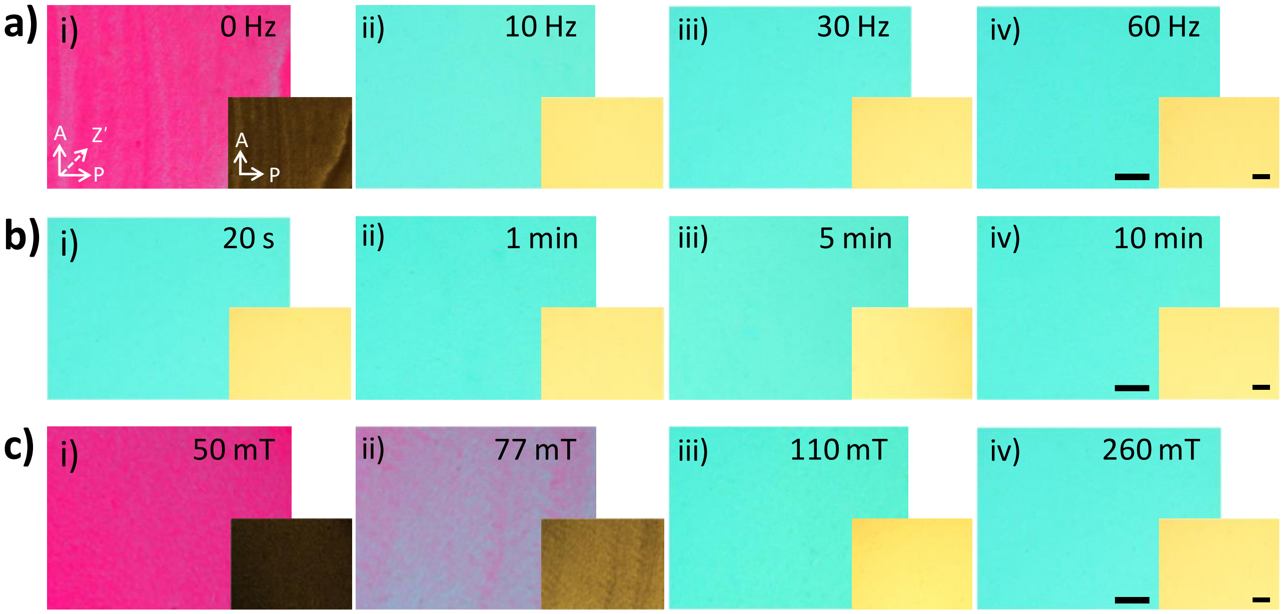

Figure S9. Anisotropic nanocomposite gel prepared with rotating magnetic fields under different experimental conditions. The experimental conditions were optimized by examining the birefringence of the gel with varying rotating frequency (a), action time (b) and magnetic strength (c). The experiments are designed by changing one parameter each time. The optimum was found with the following parameter set: rotating frequency of 30 Hz, action time of 20 s, intensity of magnetization of 260 mT. Scale bar: 2 mm.

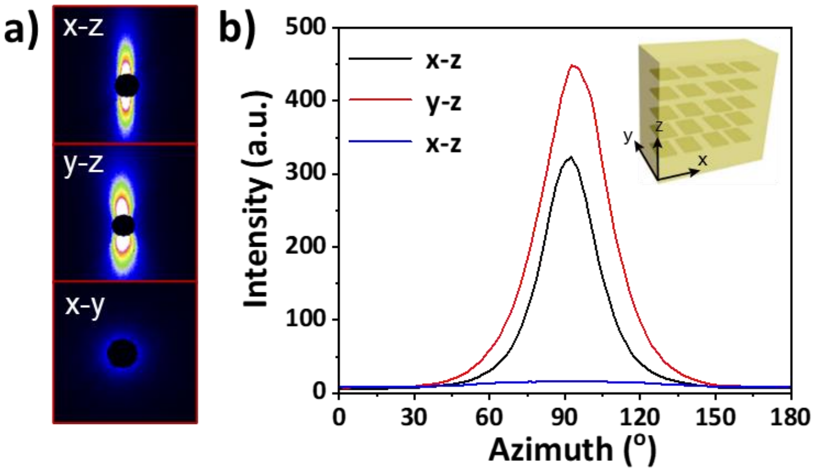

Figure S10. 2D SAXS patterns (a) and scattering intensity-azimuth plots (b) of the equilibrated hydrogel prepared with rotating magnetic fields.

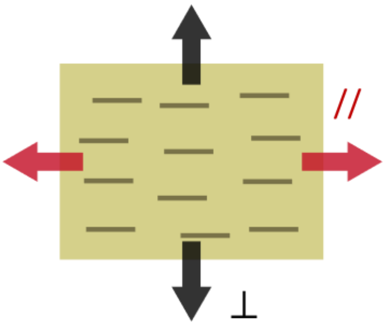

Figure S11. Schematic of the tensile directions of the equilibrated hydrogel prepared with rotating magnetic fields.

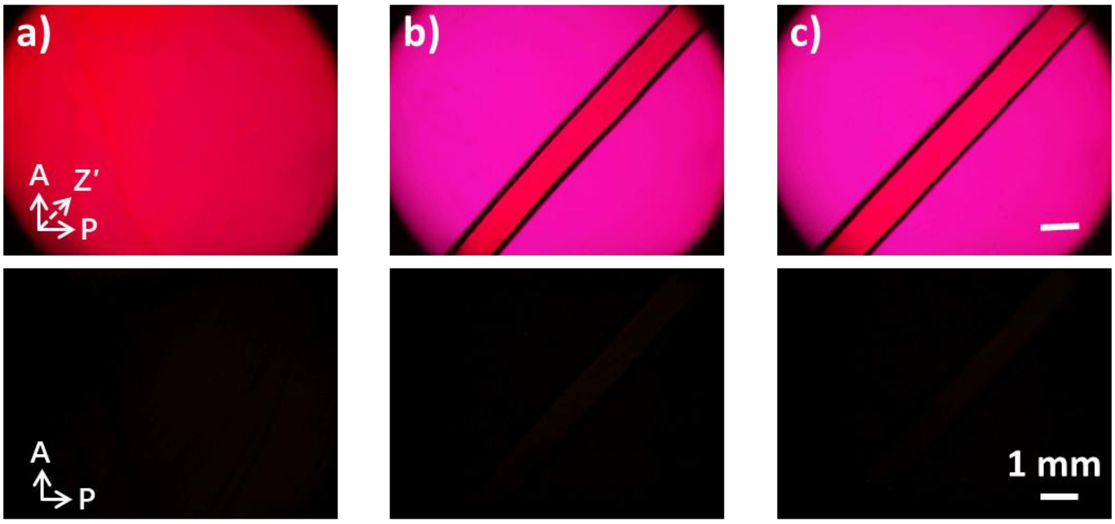

Figure S12. POM images of the MDS-containing isotropic hydrogel sheet observed from the top (a) and two orthogonal cross-sections (b, c). Thickness of gel: 1 mm. For the synthesis of isotropic gel, the reaction cell containing the precursor is oscillated with a speed of 60 rpm for 2 h at an elevated temperature to accelerate the structural relaxation of the shear-induced alignment of MDSs, which is followed by cooling to room temperature and then polymerizing under UV light irradiation.

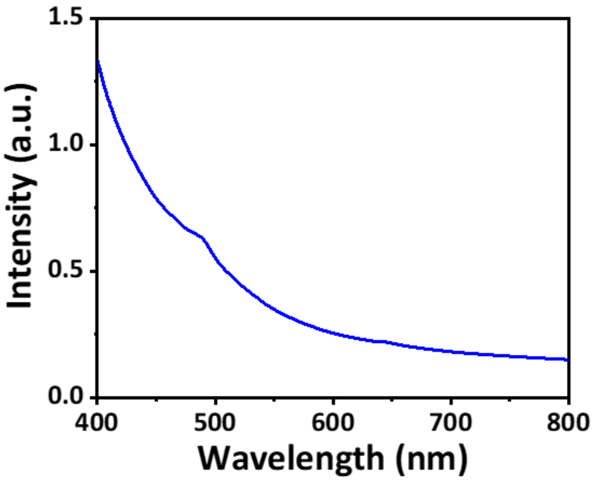

Figure S13. Absorption spectra of the hydrogel with 1 wt% of MDS prepared with rotating magnetic fields.

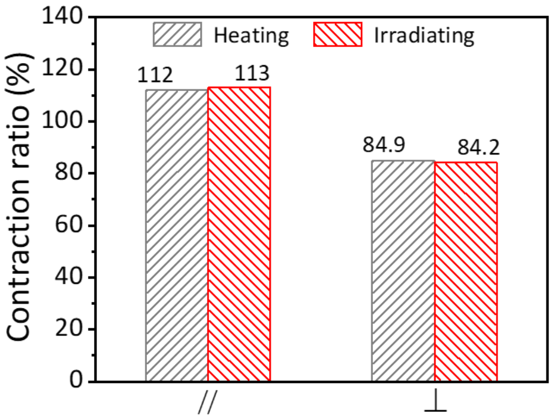

Figure S14. The maximum contraction difference in parallel (//) and perpendicular (⊥) direction incubated in warm water (40 ºC) or irradiated by a green laser.

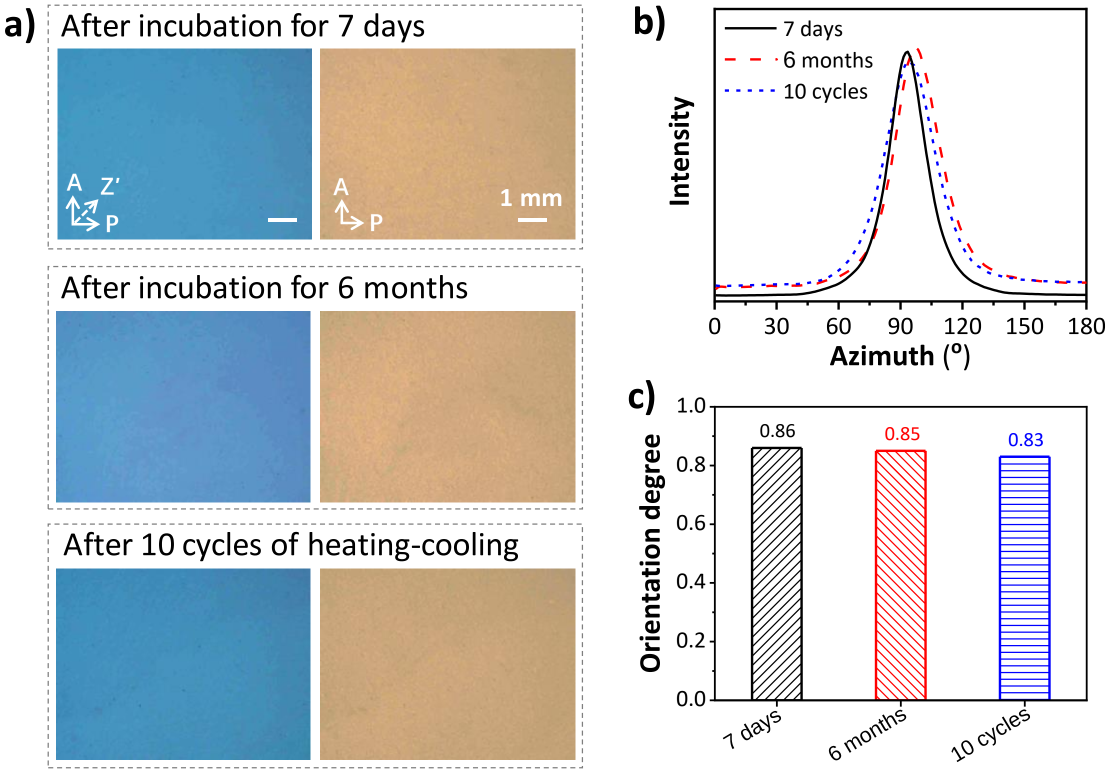

Figure S15. POM images (a), scattering intensity-azimuth plots (b), and orientation degree of MDSs in the anisotropic hydrogel after incubation in water for 7 days and 6 months, as well as after 10 cycles of heating-cooling treatments. Thickness of gel: 1 mm.

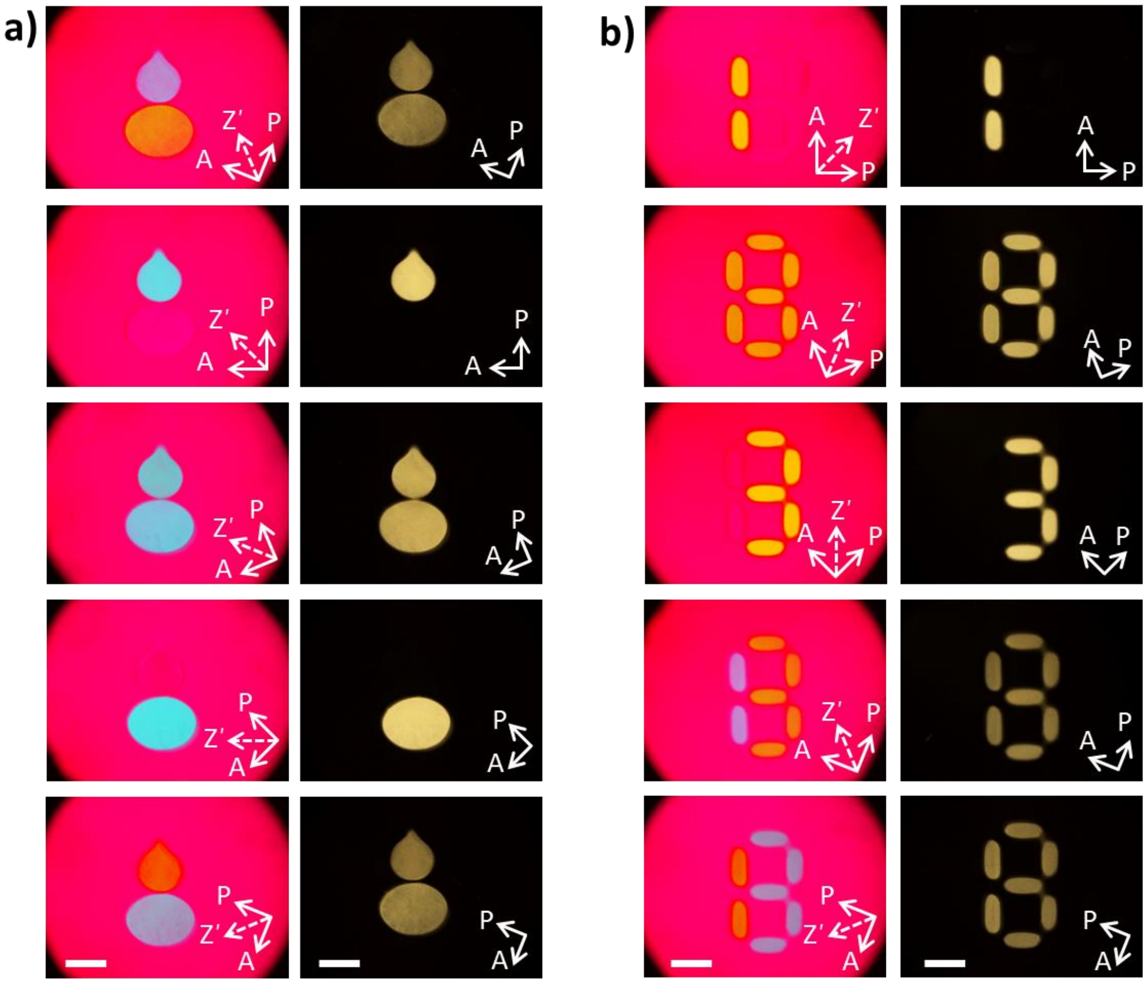

Figure S16. Additional POM images (ii) of patterned hydrogels with different alignment of MDSs at specific regions by rotating the directions of analyzer, polarizer, and tint plate. The thickness of the samples is 0.4 mm. Scale bar: 2 mm.

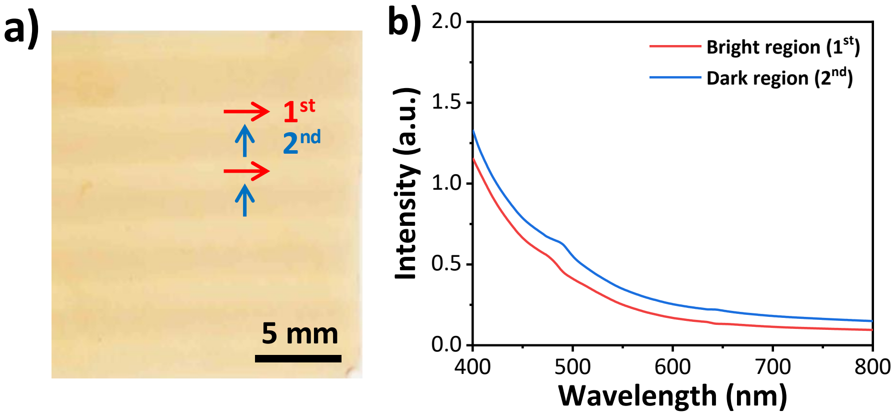

Figure S17. (a) Digital photo of stripe-patterned hydrogel under daylight. The relatively bright and dark stripes with orthogonal alignments of MDSs are synthesized multi-step photopolymerization. (b) UV-vis absorption spectra of the bright and dark regions of the patterned gel.

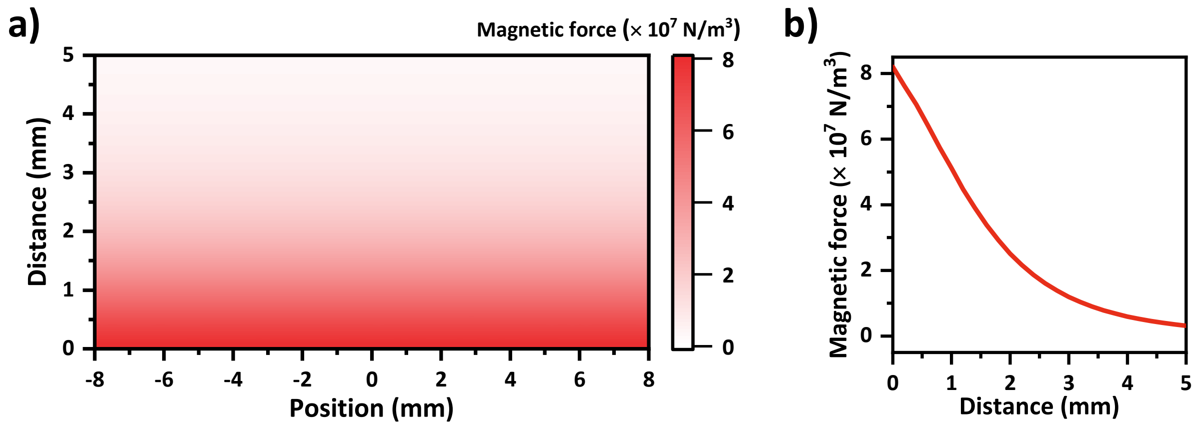

Figure S18. (a) Simulation result of the relationship between the exerted magnetic force and the position and distance of the hydrogel placed above an elongated magnet. (b) Variation of the exerted magnetic force as a function of the distance between the gel and the magnet.

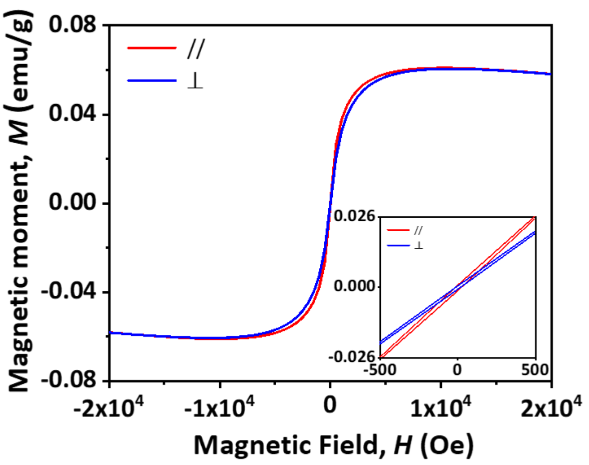

Figure S19. Magnetic hysteresis loops of gel in parallel (//) (a) and perpendicular (⊥) (b) direction.

Table S1. Comparison of responsive anisotropic hydrogels containing nanofillers as soft actuators and robots.

Material systems	Strategies for orientation of nanofillers	Achievements	Limitations	Ref.
PNIPAm hydrogel embedded with oriented titanate nanosheets (TiNSs)	Magneto-orientation by using a superconducting magnet (10 T)	Directional walking of gel with asymmetric shape by cyclic heating-cooling	The gel cannot move in the reverse direction; it took time to switch the heating and cooling.	S3
PNIPAm hydrogel embedded with oriented TiNSs and randomly dispersed AuNPs	Magneto-orientation by using a superconducting magnet (10 T)	Crawling of cylinder gel in a confined tube under scanning of a laser beam	A confined space was needed for the motion; the AuNPs might be leaked out of the gel matrix.	S4
PNIPAm hydrogel embedded with oriented alumina platelets decorated with Fe_3O_4 nanoparticles	Magneto-orientation under a rotating magnetic field using a rare-earth magnet	Programmed deformation of the gel with a bilayer structure upon heating	Deformations were slow and limited by water diffusion; it was difficult to develop anisotropic gel with sophisticated structures.	S5
Photo-responsive hydrogel embedded with oriented nickel nanowires	Magneto-orientation under static magnetic field using NdFeB rare-earth magnet	Locomotion by cooperative manipulation of light and magnetic field.	Precise manipulation of dynamic magnetic field was required; it was difficult to develop sophisticated anisotropic structures.	S6
PNIPAm hydrogel with oriented fluorohectorite nanosheets	Electro-orientation under an alternating electric field	Programmed deformation of the gel upon heating	Deformation cannot be locally and remotely triggered; it took time to switch the heating and cooling.	S7
PNIPAm hydrogel with spatially distributed MXene nanosheets	Electric field-directed electrophoresis of nanosheets	Programmed deformation of the gel upon near-infrared light irradiation	The orientation degree of nanosheets was relatively low; locomotion had not been investigated.	S8

References

[S3] Y. S. Kim, M. Liu, Y. Ishida, Y. Ebina, M. Osada, T. Sasaki, T. Hikima, M. Takata, T. Aida, *Nat. Mater.* **2015**, *14*, 1002.

[S4] Z. Sun, Y. Yamauchi, F. Araoka, Y. S. Kim, J. Bergueiro, Y. Ishida, Y. Ebina, T. Sasaki, T. Hikima, T. Aida, *Angew. Chem. Int. Ed.* **2018**, *57*, 15772.

[S5] R. M. Erb, J. S. Sander, R. Grisch, A. R. Studart, *Nat. Commun.* **2013**, *4*, 1712.

[S6] C. Li, G. C. Lau, H. Yuan, A. Aggarwal, V. L. Dominguez, S. Liu, H. Sai, L. C. Palmer, N. A.

Sather, T. J. Pearson, D. E. Freedman, P. K. Amiri, M. O. de la Cruz, S. I. Stupp, *Sci. Robot.* **2020**, *5*, eabb9822.
[S7] T. Inadomi, K. Urayama, N. Miyamoto, *ACS Appl. Polym. Mater.* **2022**, DOI: 10.1021/acsapm.2c00103.
[S8] P. Xue, K. Bisoyi, Y. Chen, H. Zeng, J. Yang, X. Yang, P. Lv, X. Zhang, A. Priimagi, L. Wang, X. Xu, Q. Li, *Angew. Chem. Int. Ed.* **2021**, *60*, 3390.

Legends for supplementary movies

Movie S1. Inefficient walking of the patterned hydrogel strip under scanning light without the magnetic force. Gel dimensions: 15 mm × 5 mm × 0.6 mm; light intensity: 2.34 W/cm^2; scanning speed: 1 mm/s. Movie speed, 5×.

Movie S2. Walking of the patterned hydrogel strip under scanning light with moderate magnetic force. Gel dimensions: 15 mm × 5 mm × 0.6 mm; light intensity: 2.34 W/cm^2; scanning speed: 1 mm/s. Movie speed, 5×.

Movie S3. Walking of the patterned hydrogel strip under light scanning with large magnetic force. Gel dimensions: 15 mm × 5 mm × 0.6 mm; light intensity: 2.34 W/cm^2; scanning speed: 1 mm/s. Movie speed, 5×.
